# Two LEAFY homologs regulate floral patterning and development without affecting flowering time in kiwifruit

**DOI:** 10.1093/jxb/erag092

**Published:** 2026-02-19

**Authors:** Yongyan Peng, Charlotte Voogd, Cecilia H Deng, Ria Rebstock, Mikaela Douglas, Bo Yang, Tianchi Wang, Andrew C Allan, Erika Varkonyi-Gasic, Joanna Putterill

**Affiliations:** Plant and Food Research Group, New Zealand Institute for Bioeconomy Science, Mt Albert, Auckland 1025, New Zealand; School of Biological Sciences, University of Auckland, Private Bag 92019, Auckland 1142, New Zealand; Plant and Food Research Group, New Zealand Institute for Bioeconomy Science, Mt Albert, Auckland 1025, New Zealand; Plant and Food Research Group, New Zealand Institute for Bioeconomy Science, Mt Albert, Auckland 1025, New Zealand; Plant and Food Research Group, New Zealand Institute for Bioeconomy Science, Mt Albert, Auckland 1025, New Zealand; Plant and Food Research Group, New Zealand Institute for Bioeconomy Science, Mt Albert, Auckland 1025, New Zealand; Plant and Food Research Group, New Zealand Institute for Bioeconomy Science, Mt Albert, Auckland 1025, New Zealand; Plant and Food Research Group, New Zealand Institute for Bioeconomy Science, Mt Albert, Auckland 1025, New Zealand; Plant and Food Research Group, New Zealand Institute for Bioeconomy Science, Mt Albert, Auckland 1025, New Zealand; School of Biological Sciences, University of Auckland, Private Bag 92019, Auckland 1142, New Zealand; Plant and Food Research Group, New Zealand Institute for Bioeconomy Science, Mt Albert, Auckland 1025, New Zealand; School of Biological Sciences, University of Auckland, Private Bag 92019, Auckland 1142, New Zealand; University College Dublin, Ireland

**Keywords:** *Actinidia chinensis*, floral organ development, flowering, gene editing, kiwifruit, LEAFY, LFY1, LFY2

## Abstract

*Actinidia chinensis* (kiwifruit) are economically important fruit-bearing woody perennial vines but with a long juvenile phase, which slows plant breeding efforts. *LEAFY* (*LFY*) encodes a plant-specific transcription factor central to the reproductive pathway in many plants and its overexpression can accelerate flowering, including in some trees. We identified two kiwifruit *LFY* genes that are expressed in actively growing apical buds. Overexpression of *AcLFY1* or *AcLFY2* promoted flowering in Arabidopsis, but did not accelerate flowering in kiwifruit. Instead, branching at the lower nodes and changes in leaf morphology were observed. We used CRISPR-Cas9 targeted mutagenesis of *LFY1* and/or *LFY2* in a fast-flowering hermaphrodite kiwifruit background to generate single- and double-mutants, which flowered at the same time as control lines. However, whilst single-mutants developed normal flowers, double-knockout mutations had severe effects on floral patterning and floral organ development. Petals and stamens were strongly affected, impacting on self-pollination, and fruit size and shape. RNA-seq showed that the two *LFY* genes were differentially regulated via the expression of *CENTRORADIALIS* genes that encode floral repressors. Our results suggest that the *LFY* genes control plant architecture and contribute together to floral organ development and floral patterning, which has implications for successful pollination and fruit set in kiwifruit.

## Teaser

Gene editing and overexpression show that *LEAFY* genes control branching and floral organ development but not flowering time in kiwifruit, unlike the flowering-time function proposed in other plants.

## Introduction

Flowering time is an important trait that determines reproductive success and is key to productivity in plants (Jung and Müller, 2009). Environmental and intrinsic factors trigger the plant to initiate the transition from vegetative to reproductive growth, leading to activation of floral meristem identity genes and then floral organ identity genes to determine the formation and patterning of flowers (Fornara *et al*., 2010). In Arabidopsis, flowering is triggered via elevation of the expression of floral integrators such as *FLOWERING LOCUS T* (*FT*), *SUPPRESSOR OF OVEREXPRESSION OF CONSTANS1* (*SOC1*), and *AGAMOUS-LIKE 24* (*AGL24*) to promote flowering (Fornara *et al*., 2010). The FT protein is synthesised in leaves and travels via the phloem to the shoot apical meristem where it forms a florigen activation complex (FAC) with the bZIP transcription factor FD and 14-3-3 proteins to switch on the floral development programme (Putterill and Varkonyi-Gasic, 2016). The FAC activates *SOC1*, which then in turn activates *FRUITFULL* (*FUL*), *AGL24*, *LEAFY* (*LFY*), and *APETALA 1* (*AP1*) to promote flowering. *LFY* is under dual regulation by the floral activator FT and the floral repressor TERMINAL FLOWER 1 (TFL1), both acting through FD, which binds to the bZIP binding sites located in the second exon of the *LFY* gene (Collani *et al*., 2019; Zhu *et al*., 2020).

*LFY* was first discovered to regulate flower development via study of the mutants *lfy* (in Arabidopsis) and *floricaula* (in *Antirrhinum majus*), which develop leafy shoots or inflorescences instead of single flowers, and by the observation that overexpression of *LFY* often accelerates flowering in annuals such as Arabidopsis and *Oryza sativa* (rice), and perennials such as *Populus* (poplar) hybrids and *Citrus sinensis* (sweet orange) (Coen *et al*., 1990; Weigel *et al*., 1992; Weigel and Nilsson, 1995; He *et al*., 2000; Peña *et al*., 2001). LFY is a transcription factor that functions to promote floral meristem identity and activate specific sets of floral organ identity genes in the ABCE flowering model via interaction with specific co-factors (Bowman and Moyroud, 2024). There are four classes of function in the ABCE model, each consisting of multiple genes that determine the formation of distinct domains of floral whorls (Weigel and Meyerowitz, 1994; Pelaz *et al*., 2000). LFY promotes the expression of the A-class gene *AP1* in whorls 1 and 2 to form sepals and petals, respectively, and interacts with UNUSUAL FLORAL ORGANS (UFO) to promote the B-class genes *AP3* and *PISTILLATA* (*PI*) in whorls 2 and 3 (petals and stamens) and with WUSCHEL (WUS) to promote expression of the C-class gene *AGAMOUS* (*AG*) in whorls 3 and 4 (stamens and carpels) (Lee *et al*., 1997; Lamb *et al*., 2002; Benlloch *et al*., 2011; Moyroud *et al*., 2011; Winter *et al*., 2011; Rieu *et al.*, 2023). Genome-wide investigations of DNA-binding of LFY in Arabidopsis have revealed several direct targets that are involved in the control of floral meristem identity, organ and flower development, as well as controlling expression of genes involved in hormone responses and biotic stimulus responses (Saddic *et al*., 2006; Winter *et al*., 2011; Goslin *et al.*, 2017). These targets include the A, B, C, and E genes (Lamb *et al*., 2002; Winter *et al*., 2011; Jin *et al*., 2021). More recently, LFY has been regarded as a pioneer transcription factor in plants due to its ability to bind methylated and non-methylated DNA, open up chromatin, and reprogramme floral fate (Jin *et al*., 2021; Lai *et al.,* 2021). In addition to direct binding to the *cis*-regulatory regions of the floral organ identity genes, LFY can bind to a nucleosome-occupied target site and displace H1 linker histones by recruiting chromatin re-modelers. This activates transcription of *AP1* and other transcription factors to promote floral fate.

*Actinidia chinensis* (kiwifruit) are woody perennial vines that are dioecious with separate male and female plants, and after pollination the female flowers produce high-value, nutritious, and tasty fruit (Ferguson, 2016). One of the challenges of kiwifruit crop improvement by plant breeding is the long juvenile phase, typically of five years, during which the vine does not bear fruit (Ferguson, 2016). Dominant early-flowering provides an alternative plant breeding strategy, as demonstrated in apple and pear where transgenic overexpression of a MADS-box transcription factor from *Betula pendula* (*MADS4*) accelerates flowering and shortens breeding cycles from 10 years to less than two years (Flachowsky *et al*., 2007; Tomes *et al*., 2023). Similar strategies have been less successful in kiwifruit. Previous studies in kiwifruit have identified MADS box genes including *AP1*, *PI*, *AG*, and *SEPALLATA* (*SEP1*) that are involved in the specification of floral organs, as well as homologs of the flowering-time genes *SOC1*, *AGL24*, and *SHORT VEGETATIVE PHASE*-like (*SVP*) (Varkonyi-Gasic *et al*., 2011; Wu *et al*., 2012, 2014; Voogd *et al*., 2015; Ye *et al.,* 2022); however, overexpression in kiwifruit has resulted in altered bud-break time without early flowering (Voogd *et al.*, 2015; Wu *et al.*, 2017, 2018). Furthermore, overexpression of either of the three kiwifruit *FT* genes results in non-viable *in vitro* flowering (Voogd *et al*., 2015, 2017; Moss *et al*., 2018). More progress has been achieved with overexpression of *AcFT1* fused with six copies of the hemagglutinin (HA) tag, which occasionally results in viable dominant early-flowering kiwifruit (Herath *et al*., 2023). Therefore, identifying a more reliable gene to drive dominant flowering would be beneficial to accelerate kiwifruit breeding, while providing better understanding of the genetic regulation of flowering in this woody perennial vine.

The role of LFY in regulation of flower development and accelerated flowering appears to be conserved across various annual and perennial species. The *LFY* loci have been shown to be located in highly conserved genomic regions in different angiosperm genomes, and *LFY* homologs have remained as a single-copy gene in most angiosperm species (Gao *et al*., 2019). The ability of LFY to regulate and accelerate flowering, and the presence of two gene copies in kiwifruit (*LFY1* and *LFY2*) prompted our interest in exploring their roles. In this study, we have used overexpression in Arabidopsis and in wild-type kiwifruit, and CRISPR-Cas9 gene-editing in the fast-flowering hermaphrodite kiwifruit *cen4 sygl* double-mutant (Varkonyi-Gasic *et al*., 2021). Promoter transactivation assays were used to identify possible upstream regulators of *LFY*. RNA-sequencing analysis of kiwifruit shoot apices mis-expressing *CENTRORADIALIS* (*CEN*) genes that encode floral repressors revealed that *LFY*s appear to be under regulatory control by CENs in kiwifruit. This study identifies the critical role of the two *LFY* genes in kiwifruit floral patterning and development in fast-flowering hermaphrodite kiwifruit.

## Materials and methods

### Gene cloning

A reciprocal BLAST search (Altschul *et al.*, 1990) using Arabidopsis *LFY* (AT5G61850) as a query was performed in the genome of kiwifruit *Actinidia chinensis* Red 5 (Pilkington *et al*., 2018), and this identified two kiwifruit *LFY* genes, *LFY1* (Acc17960) and *LFY2* (Acc29602). Candidate genes were identified from a previous study (Varkonyi-Gasic *et al*., 2011) and some were identified by BLASTP (Altschul *et al*., 1990) with genes of known function in the *A. chinensis* Red 5 genome. Full-length coding sequences of kiwifruit *LFY1* and *LFY2* were amplified from cDNA of kiwifruit shoot apices (for primers see Supplementary Table S1) and cloned into the pSAK277 vector under the control of the *CaMV35S* promoter using an In-Fusion HD Cloning Kit (TaKaRa Bio). The following kiwifruit genes were expressed under the *CaMV35S* promoter using the constructs described by Voogd *et al*. (2015): *SOC1a* (Acc16764), *SOC1b* (Acc33776), *SOC1c* (Acc19959), *SOC1d* (Acc15737), *SOC1e* (Acc14600), *SOC1f* (Acc07652), *SOC1g* (Acc16017), *SOC1h* (Acc03527), and *SOCi* (Acc16642). The following kiwifruit genes were expressed under the *CaMV35S* promoter using the constructs described by Varkonyi-Gasic *et al*. (2011): *FUL* (Acc26639), *SEP3* (Acc32725), *SEP4* (Acc26640), and *AP1* (Acc04040) and *FD* (Acc05237) was obtained described by Varkonyi-Gasic *et al.* (2013) and *CEN4* (Acc21519) was described by Voogd *et al*. (2017). The following additional candidate genes were also identified using BLASTP: *WUS* (Acc08359, Acc12050, Acc16985), *AP1-1* (Acc14105), *AP1-2* (Acc18368), *FD1* (Acc04527), and *FD2* (Acc23536), and were amplified from cDNA from wild-type kiwifruit shoot apices and cloned into the pHEX2 vector under the control of the *CaMV35S* promoter. The construct *35S:CEN4-6xHA* was generated by fusing the coding sequence of kiwifruit *CEN4* (Acc21519) to six copies of haemagglutinin (HA) tag via the linker sequence GGSGSGSS. The construct was synthesised by GenScript and cloned into the pHEX2 plasmid under the control of the *CaMV35S* promoter using Gateway Cloning (ThermoFisher Scientific).

### CRISPR-Cas9 gene editing and genotyping

Three gene-editing constructs were designed to target *LFY1* and *LFY2* in kiwifruit individually or simultaneously. Construct one contained three single-guide RNAs (sgRNAs) (Supplementary Table S1), targeting exon 1 and exon 2 of *LFY1*. Construct two contained three sgRNAs, targeting exon 1 and exon 2 of *LFY2*. Construct three contained all six of these guides targeting both *LFY1* and *LFY2*, and an additional sgRNA targeting a region in exon 2 that is identical in both *LFY1* and *LFY2.* The guides were designed in Geneious Prime using target selection criteria (Doench *et al*., 2014), and placed in the tandemly arrayed tRNA–gRNA construct under the control of the Arabidopsis *U6-26* promoter (Dang *et al*., 2015; Xie *et al*., 2015). The three constructs were synthesised by GenScript and cloned into pDE-KRS plasmids (Varkonyi-Gasic *et al*., 2019) containing *CaMV35S:Cas9* using Gateway Cloning. The constructed plasmids were verified by sequencing (Macrogen, South Korea) and transformed into *Agrobacterium tumefaciens* strain EHA105 by electroporation for kiwifruit transformation. PCR amplification was performed to genotype the CRISPR-Cas9 edited products using a Phire Plant Direct PCR Kit (ThermoFisher Scientific) following the manufacturer’s protocol. PCR products were sent for sequencing (Macrogen) for initial screening. For subsequent edit verification in plants, PCR products were cloned into the pJET1.2/blunt cloning vector supplied in the CloneJET PCR Cloning Kit (ThermoFisher Scientific), and at least six clones of each line were analysed.

### Plant material, transformation, and growth

For overexpression of *AcLFY* in Arabidopsis Col-0, Arabidopsis plants were grown in a controlled growth room under 16/8 h light/dark cycle (200 μmol m^–2^ s^–1^) at 22 °C. Arabidopsis transformation using the pSAK277 overexpression vector constructs with *AcLFY1*, *AcLFY2*, and *GUS* (control), all driven by the *35S* promoter, was performed by floral dip using *Agrobacterium* strain GV3101 (Clough and Bent, 1998). The seeds were collected and surface-sterilised in 70% ethanol for 2 min, followed by 10% sodium hypochlorite for 10 min. Seeds were rinsed with sterilised distilled water and kept in the dark at 4 °C for 2 d. The seeds were sown on half-strength Murashige and Skoog (MS) selection media containing 1% sucrose, 50 mg l^–1^ kanamycin, and 300 mg l^–1^ Timentin^®^ and maintained in a tissue-culture growth room under 16/8 h light/dark with cool white fluorescent light (35–45 µmol m^−2^ s^−1^) at 23 °C. The transgenic T1 seedlings were transferred to soil in a controlled growth room under the same growth conditions described above. A total of 13 *AcLFY1*- overexpression lines, 36 *AcLFY2*-overexpression lines, and nine *GUS*-overexpression lines were generated. Their flowering times were evaluated by counting the numbers of rosette leaves at floral transition (bolting). Two-sample *t*-tests were performed to identify significant differences between groups.

For *A. tumefaciens-*mediated transformation (Wang *et al.,* 2007), two kiwifruit lines were used, namely female Red9 (wild-type) and the fast-flowering hermaphrodite *cen4 sygl* double-mutant, which is the kanamycin-sensitive progeny of the *A. chinensis cen4 sygl* line 7 previously reported by Varkonyi-Gasic *et al.,* 2021). These were obtained from *in vitro* collections held at Plant & Food Research, Auckland, New Zealand, and plants were under 16/8 h light/dark with cool white fluorescent light (35–45 µmol m^−2^ s^−1^) at 23 °C.

The pSAK277 overexpression vector constructs with *AcLFY1* and *AcLFY2* and the pSAK277 empty vector were transformed into wild-type kiwifruit (Wang *et al*., 2007). This resulted in 23 independent *AcLFY1*-overexpression lines, six *AcLFY2*-overexpression lines, and five pSAK277 empty-vector lines being generated. The same vectors were also transformed into the fast-flowering hermaphrodite kiwifruit, resulting in seven independent *AcLFY1*-overexpression lines, seven *AcLFY2*-overexpression lines, and 11 empty-vector control lines being generated. Rooted transgenic T0 kiwifruit plants were transferred into soil and kept in the controlled growth room, then transferred to a controlled acclimatisation growth room with 18/30 °C minimum/maximum night/day temperature a 14/10 h light/dark photoperiod cycle. The transformed fast-flowering hermaphrodite kiwifruit plants stayed in this room while the transformed wild-type (‘Red9’) background plants with more than 30 leaves were transferred to the glasshouse and grown under ambient conditions for 3 years with 3 months winter chilling every year.

For CRISPR-Cas9 gene editing of *LFY* in kiwifruit, the three constructed pDE-KRS plasmids described above (targeting *AcLFY1*, *AcLFY2*, and both simultaneously) were transformed into the fast-flowering hermaphrodite kiwifruit, and generated a total of 37 independent lines. Genotyping by PCR (as described above) identified 16 lines with no editing events and these are referred to as non-edited controls. These rooted plants were transferred into soil and were cultivated using the same process and growth conditions as described above. The flowering times of the transformed kiwifruit lines were evaluated by counting the numbers of leaves at flower transition, and two-sample *t*-tests were performed to identify significant differences between groups.

For transcriptomic analysis, the pHEX2 overexpression vector construct with *AcCEN4-6×HA* and the pHEX2 empty vector control were transformed into wild-type kiwifruit (Red9) and ∼50 regenerated transformant shoots from each were sub-cultured and maintained on MS media containing, 0.2 mg l^–1^ zeatin, 2% sucrose, 0.7% agar, 50 mg l^–1^ kanamycin, 300 mg l^–1^ timentin, pH 5.8. Shoot apices from ∼15 shoots were pooled as one biological replicate, and three biological replicates were sampled from each construct. The presence of the *AcCEN4-6×HA* transgene was confirmed by PCR genotyping. In addition, *cen cen4* fast-flowering double-mutants were generated in the kiwifruit wild-type background using CRISPR-Cas9-mediated mutagenesis. PCR genotyping identified biallelic gene edits in both *CEN* and *CEN4* (Supplementary Table S2): in the gene-editing context, the use of the term biallelic refers to mutations caused by gene editing in both alleles of the gene. Two independent *cen cen4* lines (numbers 26 and 29) that flowered at ∼15 nodes were deemed to be fast-flowering, and were introduced back into tissue culture after sterilisation. The sterilised stems were dried and placed onto MS maintenance media containing, 0.2 mg l^–1^ indolebutyric acid, 0.2 mg l^–1^ zeatin, 2% sucrose, 0.65% agar, pH 5.8. Subsequently, ∼50 shoots were sub-cultured from the two lines and maintained in tissue culture, along with ∼50 shoots sub-cultured from wild-type plants. For transcriptomics, we used biological replicates consisting of ∼15 shoot apices, with one biological replicate being taken from line 26 and two from line 29.

### Promoter analysis and dual-luciferase transient assays

Genomic DNA was extracted from the shoot apices of kiwifruit ‘Hort16A’ using a DNeasy Plant Mini Kit (Qiagen). The 2.1 kb promoter regions upstream of the ATG of *LFY1* and *LFY2* were isolated by PCR and cloned into the pGreen0800-LUC vector using BamHI/NcoI restriction cloning, placing the promoter upstream of the luciferase reporter gene. The plasmids were transformed into *A. tumefaciens* strain GV3101 by electroporation. The promoter sequences were confirmed by sequencing (Macrogen). The potential binding motifs were screened using the Plant ChIP-seq Database (PCBase 2.0, http://pcbase.itps.ncku.edu.tw/) (Chow *et al*., 2024). The promoter-activation dual-luciferase assays were performed on leaves of 6-week-old *Nicotiana benthamiana* plants by co-infiltrating the vector carrying the promoter sequence with the overexpression vector containing the candidate genes (Hellens *et al*., 2005). One plant was treated as one biological replicate and four biological replicates were infiltrated for each co-infiltration combination. At 4 d after infiltration, four leaf discs harvested from each plant were treated as four technical replicates and were assayed using a Renilla Firefly Dual Assay kit (Targeting Systems, USA) to measure firefly luciferase (LUC) and *Renilla* luciferase (REN) activity. The promoter activities were expressed as the LUC/REN activity ratio, and two-sample *t*-tests were performed to identify significant differences between group means.

### Gene expression analysis

The expression of *LFY1* and *LFY2* in orchard-grown *A. chinensis* kiwifruit across seasons was determined using previously reported RNA-seq data (Brian *et al*., 2021; Voogd *et al*., 2022) obtained from flower, fruit, and vegetative tissues of female ‘Hort16A’, and from axillary and terminal buds of female ‘Zesy002’ (Gold3). For RT-qPCR on the gene-edited fast-flowering hermaphrodite *lfy* plants and non-edited controls, RNA was extracted from the floral buds. One biological replicate consisted of a pool of three to five floral buds, and three biological replicates were collected for each line. For the RNA-seq, RNA was extracted from the shoot apices of tissue-culture-grown kiwifruit with *AcCEN4-6×HA* and the empty-vector control, and the gene-edited *cen cen4* double-mutant and the wild-type lines. One biological replicate consisted of ∼15 shoot apices, and three biological replicates were collected for each group.

RNA extraction was performed using a Spectrum Plant Total RNA Kit (Sigma-Aldrich) following the manufacturer’s protocol. For RT-qPCR analysis, the RNA was reverse-transcribed into cDNA using a QuantiTect Reverse Transcription Kit (Qiagen) following the manufacturer's protocol. Gene-specific oligonucleotide primers for kiwifruit *AP1* (Acc04040), *AP1-1* (Acc14105), *AP1-2* (Acc18368), *AP2* (Acc06022), *PI* (Acc05042), *AP3-2* (Acc23601), *AG* (Acc21359), *SEP1* (Acc04041), *SEP3* (Acc32725), *ACTIN* (Acc05529), and *EF1a* (Acc24787) were designed using Geneious Prime and are summarised in Supplementary Table S1. RT-qPCR was carried out using a LightCycler 480 system with LightCycler^®^ 480 SYBR Green I Master Mix (Roche) as described previously (Peng *et al*., 2020). Four technical replicates each containing 5 µl reaction volume were run with the non-template control and water control. The data output was analysed by the LightCycler480 software v.1.5 using the target/reference ratio to compare the expression level of the target genes normalised to the reference genes *ACTIN* and *EF1a*. Two-sample *t*-tests were performed to determined significant differences between groups.

For RNA-seq, the quantity and quality of samples were checked using an Agilent Bioanalyzer 2100 with the RIN score above 7. RNA samples were sent to Novogene (Hong Kong) for directional strand-specific mRNA library preparation and Illumina NovaSeq 6000 PE150 sequencing. Data quality checking was performed with FastQC v.0.11.9 (https://www.bioinformatics.babraham.ac.uk/projects/fastqc/) and trimmed with TrimGalore V.0.6.6 (https://zenodo.org/records/7598955). Processed reads were mapped to the *A. chinensis* var. *chinensis* Red5_PS1_1.69.0 genome (Pilkington *et al*., 2018) using STAR v2.6.1d (Dobin *et al*., 2013). The alignment quality was checked and visualised with Qualimap v2.2.2 (García-Alcalde *et al*., 2012; Okonechnikov *et al.*, 2016). PCR artefacts were checked with dupRadar v1.18.0 (Sayols *et al*., 2016), gene expression quantification for each sample including read counts, FPKM, and TPM was generated using Salmon v1.3.0 (Patro *et al.,* 2017) and are presented as fragments per kilobase of transcript per million reads (FPKM) of three biological replicates. Differentially expressed genes (DEGs) were identified using DESeq2 v1.28.0 (Anders and Huber, 2010) based on the raw read counts from each sample. DEGs were selected based on a cut-off of log_2_fold-change ≥ 1 and ≤ –1 and adjusted *P*-value ≤0.05. Gene Ontology (GO) enrichment analysis of DEGs that were significantly over-represented in biological processes was performed using ShinyGO v0.82 (Ge *et al*., 2020) with a false-discovery rate *P*-value cut-off of 0.05.

### Kiwifruit seed germination

The *lfy* single-mutants were capable of self-pollination whereas the *lfy* double-mutants were cross-pollinated by applying wild-type kiwifruit pollen to the flower styles using a paintbrush. Immature seeds were collected from the fruit at ∼60 days after full bloom (DAFB) and mature seeds were collected from fruit at ∼100 DAFB. The seeds were surface-sterilised in 70% ethanol for 2 min, then with 7.5% sodium hypochlorite and a drop of Triton-X 100 for 10 min. After thoroughly rinsing with sterile distilled water, seeds were cultured in the tissue-culture growth room set at 23 °C as described above. Seeds from the fast-flowering hermaphrodite kiwifruit used for transformation was also used as a control for germination. For seed viability testing, embryos were excised from water-primed seed placed in a 1% tetrazolium solution (pH 6.5–7.5), covered with tinfoil, and left overnight at room temperature. The embryos were then examined under a dissecting microscope to assess red staining indicative of respiring cells.

### SEM

Closed and open floral buds from two independent kiwifruit *lfy1 lfy2* double-mutant lines and non-edited controls were fixed in 2% paraformaldehyde and 2.5% glutaraldehyde in a 0.1 M phosphate buffer for at least one week. The buds were rinsed in 0.1 M phosphate buffer, longitudinally sectioned using a scalpel blade, then dehydrated through an ethanol series starting at 50% and finishing with two changes of 100% ethanol, for 1 h each step. They were then subjected to critical-point drying using a Leica EM CPD300 to carry out 24 exchanges of liquid CO_2_ over 4 h. Dried buds were mounted onto stubs using sticky carbon tape and coated in gold for 3 min using the diffuse sputtering mode in a Leica EM ACE200 Sputter Coater. Imaging was carried out using a CLARA field-emission SEM (Tescan, Brno, Czechia) at 5 keV and 30 pA for surface-sensitive imaging, with a spot size of 18 nm and working distance of 86 mm to visualise the whole bud.

## Results

### Two kiwifruit *LFY* homologs are expressed in floral and apical buds during spring and summer

**Fig. 1.**
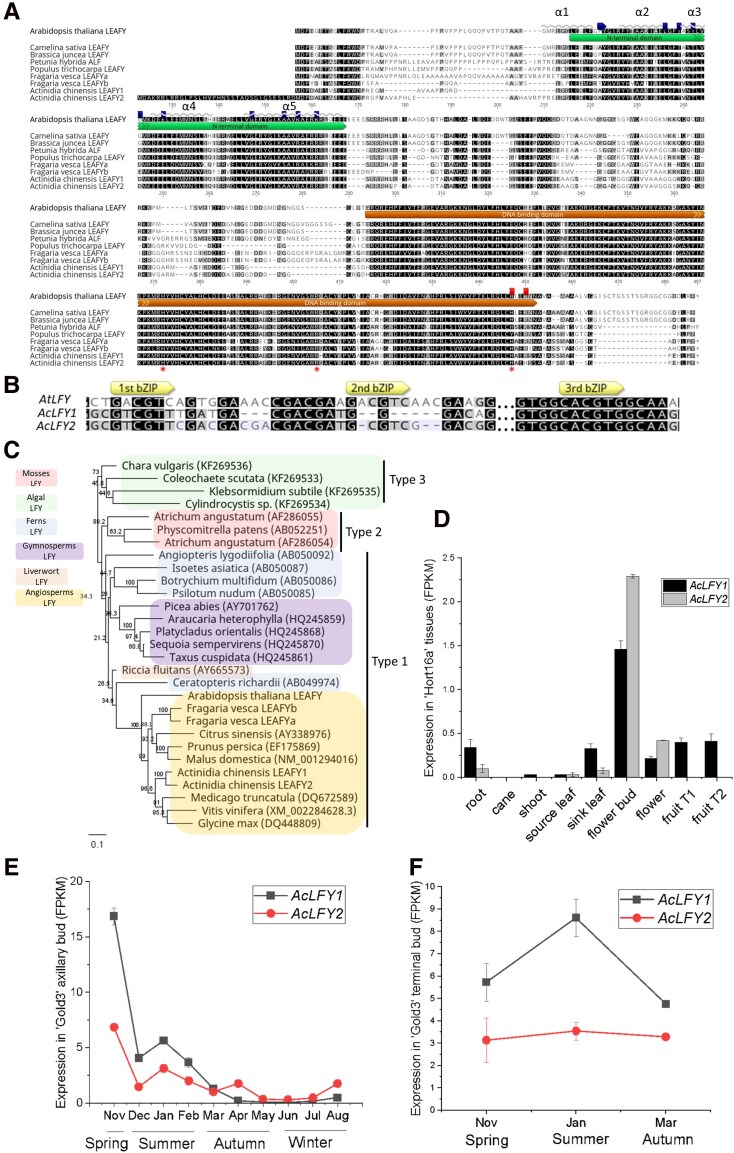
Identification of two kiwifruit *LFY* genes and their expression patterns. (A) Amino acid sequence alignment of predicted LFY proteins. The N-terminal domain and DNA-binding domain are labelled. The three critical amino acid sites 312H, 345R, and 387H for type 1 DNA motif-binding are labelled with asterisks. The numbering of residues refers to the Arabidopsis LFY sequence. Alpha helices are indicated by the diagrammatic coils. Small boxes indicate the key interaction residues of the Sterile Alpha Motif oligomerisation domain in the N-terminal domain and small boxes in the DNA-binding domain indicate the key residues involved in interactions between DNA-binding domain monomers (Sayou *et al*., 2014, 2016). (B) Transcription factor bZIP binding motifs in exon 2 of Arabidopsis *AtLFY* and kiwifruit *AcLFY1* and *AcLFY2* identified from Zhu *et al.* (2020). (C) Phylogenetic tree with the representative LFY proteins identified from the six divisions of the plant kingdom, based on Sayou *et al.* (2014). The amino acid sequences were aligned using MUSCLE alignment in Geneious Prime. The genetic distance was calculated by the Jukes–Cantor model, and the tree was built using the neighbor-joining method with 1000 bootstrap replicates in Geneious Prime. The classification into three types is based on the type of DNA-binding motifs. (D) Gene expression analysis of the two kiwifruit *LFY* genes in various vegetative, fruits and flower tissue from ‘Hort16A’ female plants collected from orchards in New Zealand. Expression data are from previously published RNA-seq studies (Brian *et al.*, 2021; Voogd *et al*., 2022) and expressed as fragments per kilobase per million (FPKM). Data are means (±SE) of three biological replicates. Fruit T1 and T2 represent fruit at 20 d and 40 d after anthesis, respectively. (E, F) Gene expression analysis of the two kiwifruit *LFY* genes in different tissues of ‘Gold3’ orchard-grown plants at intervals from spring to winter in New Zealand (Brian *et al.*, 2021; Voogd *et al*., 2022). (E) Expression in axillary buds. Data are means (±SE) of three biological replicates, except for November that has two biological replicates. (F) Expression in terminal buds. Data are means (±SE) of two biological replicates for November and January, and a single replicate for March.

Two *LFY* homologs were identified in kiwifruit, named *LFY1* (Acc17960) and *LFY2* (Acc29602), and they encode proteins that share 88.2% identity and are 63.7% and 65% identical to Arabidopsis LFY, respectively (Fig. 1A). The predicted amino acid sequence of LFY2 includes an extended N-terminus of 34 amino acids upstream of the conserved methionine shared with LFY1; however, we did not investigate further whether it is indeed part of the translated LFY2 protein or whether it has functional significance. Both LFY1 and LFY2 possess a sterile alpha motif (SAM) oligomerisation domain in the N-terminal domain and the DNA-binding domain, and the key amino acid residues in these domains are highly conserved. The SAM domain mediates and allows LFY to oligomerise and access closed chromatin regions (Sayou *et al*., 2016). The three putative bZIP-binding sites in the second exon of Arabidopsis *LFY* are crucial for the recruitment of TFL1/FD (Zhu *et al*., 2020) and we examined them in the kiwifruit *LFY* genes. We found that the first bZIP C-box (GACGTC) motif was partially conserved with variation at A2 and C6 in the *AcLFY*s compared with *AtLFY* (Fig. 1B). The second bZIP C-box motif was partially conserved in the second exon of *AcLFY2* (GCGTC) but it was absent in the second exon of *AcLFY1*. The third bZIP G-box (CACGTG) motif was conserved in both *LFY1* and *LFY2*. In terms of the type of DNA-binding motifs, phylogenetic alignment with representative LFY proteins from the six divisions of the plant kingdom classified both LFY1 and LFY2 as type 1 (Fig. 1C) (Sayou *et al*., 2014).

We next examined the expression of the two *LFY* genes in previously reported RNA-seq data from various tissues of the diploid female kiwifruit ‘Hort16A’ and from terminal and axillary buds of the tetraploid female kiwifruit Gold 3 (Brian *et al*., 2021; Voogd *et al*., 2022). Both *LFY1* and *LFY2* were predominantly expressed in flower buds (Fig. 1D), in axillary buds during the active growth stage (Fig. 1E), and in terminal buds (Fig. 1F). Highest expression for both genes was observed in the axillary buds during active growth in spring and summer (November–February, Southern Hemisphere), and expression was reduced in the inactive growth periods in autumn and winter (March–August). These expression patterns suggested that the genes might be involved in meristem development in the buds during the active growth phase.

### Overexpression of kiwifruit *LFY*s induces early, solitary flowers in Arabidopsis

**Fig. 2.**
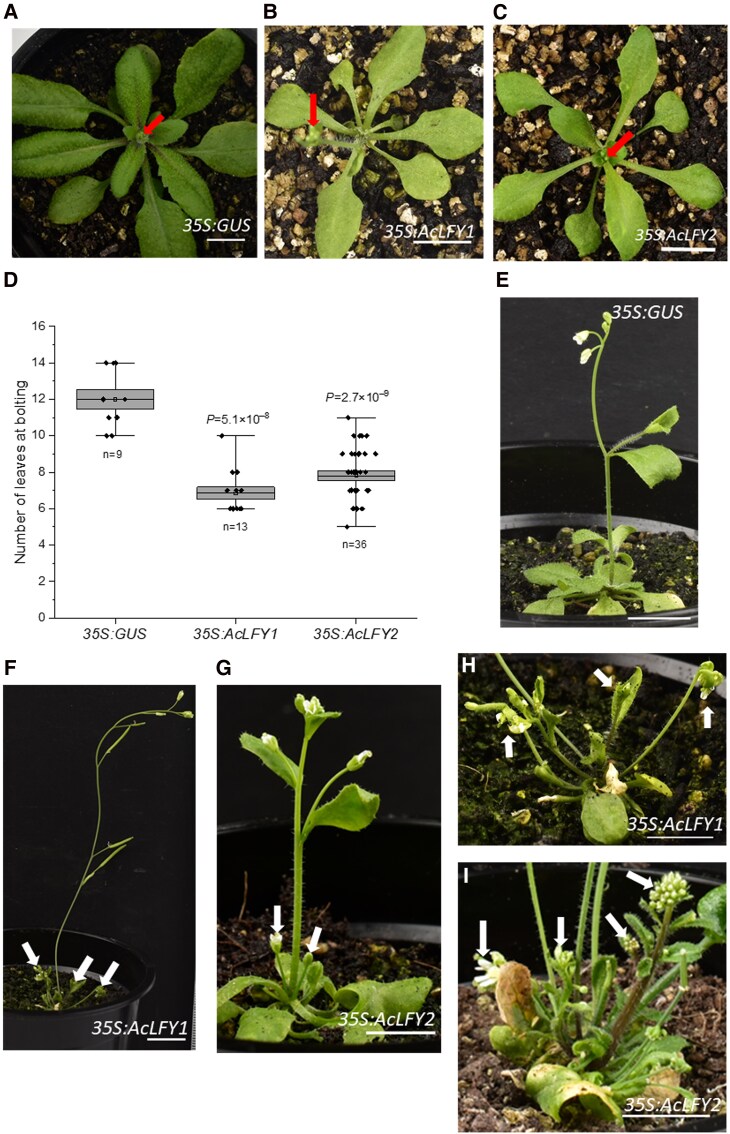
Overexpression of kiwifruit *AcLFY* genes in Arabidopsis promotes early flowering. (A–C) Representative images of Arabidopsis plants transformed with (A) *35S:GUS* (control), (B) *35S:AcLFY*1, and (C) *35S:AcLFY*2. The arrows indicate the appearance of inflorescences at the bolting stage. Scale bar&1 cm. The images are reproduced from Peng *et al.* (2025) with permission from the International Society for Horticultural Science. (D) Flowering time expressed as numbers of leaves at bolting recorded in T1 lines of the three genotypes grown in long-day conditions (16/8 h light/dark). The plots show the mean (line inside box), the standard error of the mean (box), and the maximum and minimum data points data points (whiskers). The numbers of replicates are shown together with the significant differences compared with the *35S:GUS* control, as determined using two-sample *t*-tests. (E) Arabidopsis transformed with the *35S:GUS* control, and solitary floral structures formed at the axils of the rosette leaves in the lines overexpressing (F) *35S:AcLFY1* and (G) *35S:AcLFY2* (arrows). (H, I) Close-up images of (H) the floral structures formed on the *35S:AcLFY1* line and (I) the basal floral structures that emerged at the axils of rosette leaves on the *35S:AcLFY2* line. All scale bars are 1 cm.

To begin to elucidate their potential function in flowering, the two kiwifruit *LFY*s were overexpressed in Arabidopsis. Overexpression of either gene promoted early flowering in the T1 transgenic lines compared with the *35S:GUS* control (Fig. 2A–C). On average, the *35S:LFY1* lines and *35S:LFY2* lines bolted at seven and eight rosette leaves, respectively, while the control lines bolted at 12 rosette leaves (Fig. 2D). The overexpression lines showed increased numbers of solitary flowers developed in the axils of rosette leaves, where there are usually secondary shoots (Fig. 2E–G). Different degrees of solitary flowers were observed, with some having intact floral organs (Fig. 2G) while others showed distorted sepals and petals, and indeterminate inflorescence (Fig. 2H, I). These results suggested that the kiwifruit *LFY*s have floral functions.

### Overexpression of *AcLFY*s in kiwifruit induces axillary budbreak and serrated leaves but does not promote early flowering

**Fig. 3.**
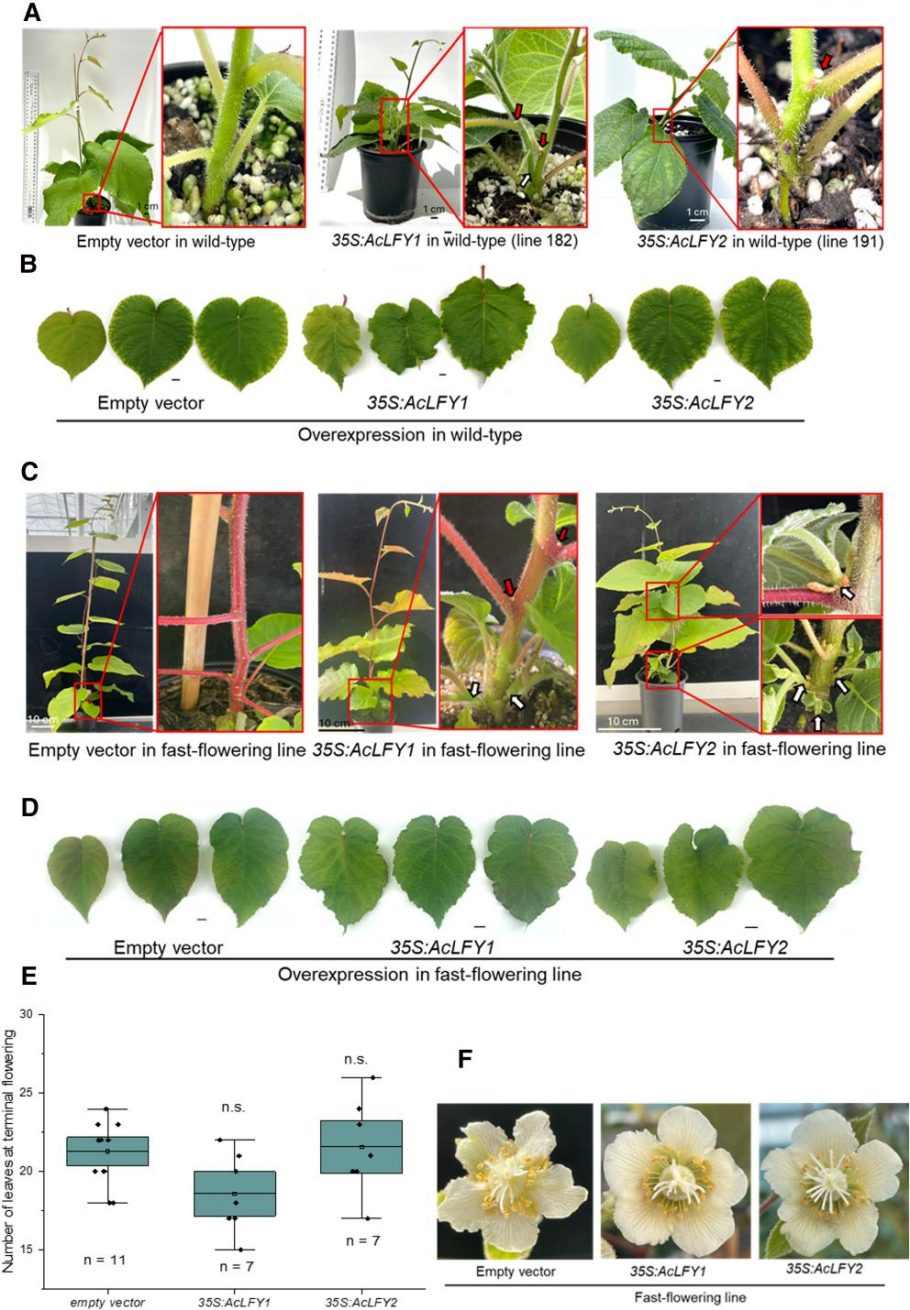
Overexpression of *LFY* genes in kiwifruit encourages lateral shoot initiation at the lower nodes and formation of serrated leaves. (A) Representative images of the empty-vector control in the wild-type (‘Red9’) and lines overexpressing *LFY1* or *LFY2*, which show activated budbreak (red arrows) and lateral shoot outgrowth (white arrow, line 182) from the primary shoot compared to the empty vector control. The boxes indicate the internode space on the primary shoot that is shown in the adjacent close-up image. (B) Images of leaves from the three genotypes, showing serrated edges in the *LFY*-overexpressing lines compared with the control. Scale bars are 1 cm. (C) Representative images of the empty-vector control in the fast-flowering hermaphrodite kiwifruit background (*cen4 sygl* double-mutant) and in lines overexpressing *LFY1* or *LFY2*, which show activated budbreak (red arrows) and lateral shoot outgrowth (white arrows) from the primary shoot compared with the control. (D) Images of leaves from the three genotypes in the fast-flowering hermaphrodite background, showing serrated edges in the *LFY*-overexpressing lines compared with the control. Scale bars are 1 cm. (E) Flowering time of the three genotypes in the fast-flowering hermaphrodite background expressed as numbers of leaves at the time of terminal flowering. The plots show the mean (line inside box), the standard error of the mean (box), and the maximum and minimum data points data points (whiskers). The numbers of replicates are shown. No significant differences (n.s.) compared with the control were found according to two-sample *t*-tests. (F) Representative images of flowers of the three genotypes in the fast-flowering hermaphrodite background.

To further examine their functions in kiwifruit, *LFY1* and *LFY2* were overexpressed in the wild-type. The 23 independent *35S:LFY1* T0 lines that were obtained showed different degrees of axillary budbreak and shoot outgrowth at ∼10 leaves, whereas the empty vector control developed a primary stem with ∼15 leaves and no obvious axillary buds (Fig. 3A; Supplementary Fig. S1). The transgenic line showing the highest *LFY1* expression had the most advanced axillary shoot outgrowth at the lower nodes while the other lines that showed more moderate expression had slower axillary budbreak and shoot outgrowth (Supplementary Fig. S1). Some of the six independent *35S:LFY2* T0 lines that were obtained showed swollen axillary buds and their shoot outgrowth was much slower than the *35S:LFY1* lines. Leaves from the *LFY1*-overexpression lines were highly serrated and curly compared with the more slightly serrated leaves of the *LFY2*-overexpression lines and the wild-type-like leaves from the empty-vector control (Fig. 3B). Kiwifruit usually takes 5 years or more to flower in controlled glasshouse conditions (Varkonyi-Gasic *et al.*, 2019). We grew *35S:LFY* plants under such conditions for 3 years with 3 months of winter chilling every year, but they showed no signs of precocious flowering.

To further investigate the role of the genes in relation to kiwifruit flowering time, *LFY1* and *LFY2* were overexpressed in the fast-flowering hermaphrodite *cen4 sygl* double-mutant background. The overexpression phenotypes were similar to those in the wild-type. Multiple axillary budbreak and shoot outgrowth at the lower nodes were observed in both the overexpression lines (Fig. 3C). The leaves showed some degrees of serration and curly edges compared with the empty-vector control (Fig. 3D). There were no significant differences in flowering time between the overexpression lines compared with the control (Fig. 3E), and the flowers from the overexpression lines showed normal phenotypes (Fig. 3F). These observations suggested that *LFY* overexpression did not promote precocious flowering in kiwifruit, regardless of the genetic background.

### Double-mutation of *lfy1 lfy2* in a fast-flowering hermaphrodite kiwifruit results in similar floral timing but altered floral form

**Fig. 4.**
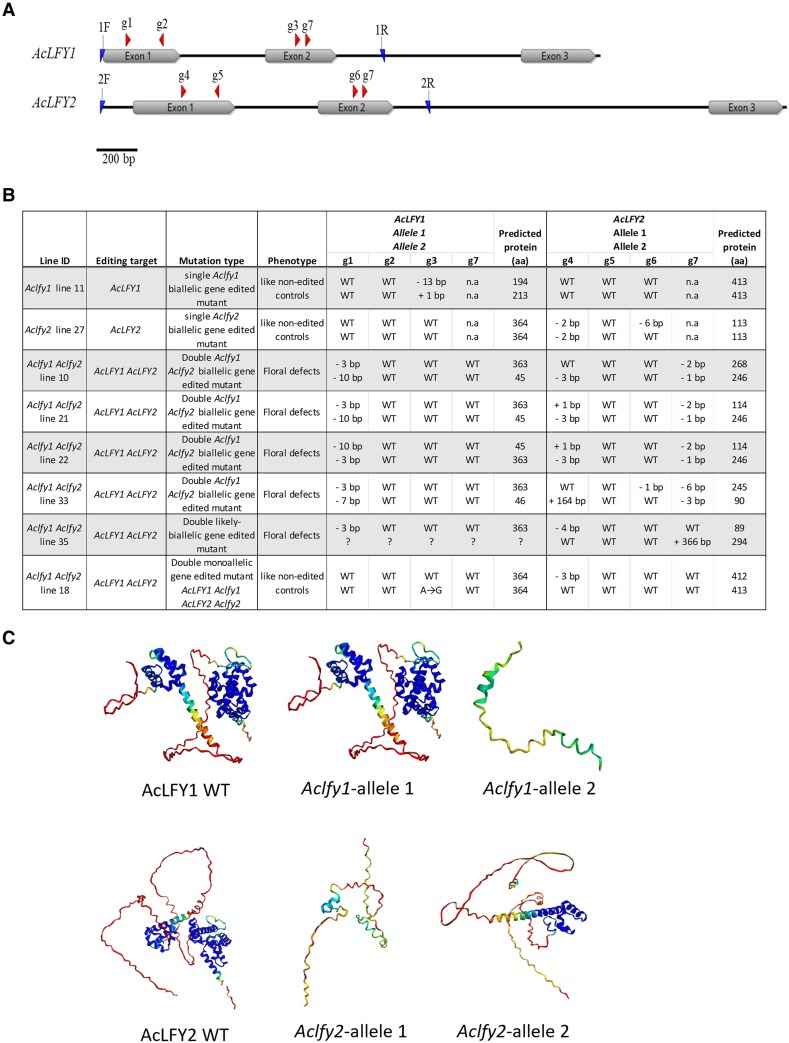
CRISPR-Cas9 gene-editing of kiwifruit *LFY1* and *LFY2* in the fast-flowering hermaphrodite background (*cen4 sygl* double-mutant). (A) Schematic representation of the two genes showing sgRNA (g) and the forward (F) and reverse (R) primers. The gene-editing constructs used g1–3 to target *LFY1*, g4–6 to target *LFY2*, and g1–7 to target both genes. (B) The editing events in T0 plants corresponding to sgRNAs targeting *LFY1* and *LFY2*; n.a. means not applicable as sgRNA7 was not used to create single-mutants. Biallelic gene edited means both alleles of a gene were gene edited, whilst monoallelic means that only one allele of a gene was edited, while the other is the wild type (WT). The full-length LFY1 protein is predicted to be 364 amino acids whilst the full-length LFY2 is predicted to be 413. Question marks indicate that the second allele apparently could not be amplified. (C) AlphaFold predictions (Jumper *et al.*, 2021) of the WT LFY1 and LFY2 proteins compared with the mutated proteins encoded by the edited alleles from the *lfy1 lfy2* double-mutant line 21.

We next used CRISPR-Cas9 gene-editing to knock out *LFY1* and *LFY2* both individually and simultaneously in a fast-flowering hermaphrodite *cen4 sygl* double-mutant kiwifruit, which was used because of the long juvenile phase of several years of kiwifruit. Sequence-specific sgRNAs were designed to target exons 1 and 2 in *LFY1* (sgRNA1, 2, 3) and in *LFY2* (sgRNA4, 5, 6) (Fig. 4A), and these were used to generate two constructs targeting each gene separately. A third construct was generated by combining sgRNA1–6 with an additional sgRNA7 designed to target a conserved region shared between *LFY1* and *LFY2* to knock out both genes. The three constructs were transformed separately and generated a total of 37 independent T0 lines. Gene-editing was confirmed by PCR genotyping to identify monoallelic (one edited and one wild-type allele) and biallelic (both alleles edited) gene-edited single- and double-mutants. Of the 37 lines, 16 were found not to be edited and are hence referred to as non-edited controls. Of the remainder, four were biallelic and three were monoallelic gene-edited *lfy1* mutants, five were biallelic and two were monoallelic gene-edited *lfy2* mutants, two were monoallelic *lfy1 lfy2* double-mutants and five were biallelic gene-edited *lfy1 lfy2* double-mutants. Most of the mutations corresponded to sgRNA1, 3, 4, 6, and 7, while sgRNA2 and 5 showed no editing activity in any of the lines (Fig. 4B). The mutations ranged from single-nucleotide insertions and deletions to a 366 nucleotide insertion, mostly creating a frameshift in the nucleotide sequence that resulted in a predicted truncated protein (Fig. 4B, C; Supplementary Fig. S2). However, the three-nucleotide deletion seen in the edited alleles of *lfy1* (in lines 10, 21, 22, 33, and 35) and *lfy2* genes (in lines 10, 18, 21, and 22) removed the first amino acid of the second alpha-helix coil in the SAM oligomerisation domain (Supplementary Fig. S2; Sayou *et al*., 2016).

Interestingly, both the *lfy1* and *lfy2* single-mutants and the *lfy1 lfy2* double-mutants flowered at a similar time as the fast-flowering hermaphrodite non-edited control lines, at ∼22–25 nodes (Supplementary Table S3). This showed that there was no requirement for *LFY* genes for the rapid flowering observed in these fast-flowering lines.

**Fig. 5.**
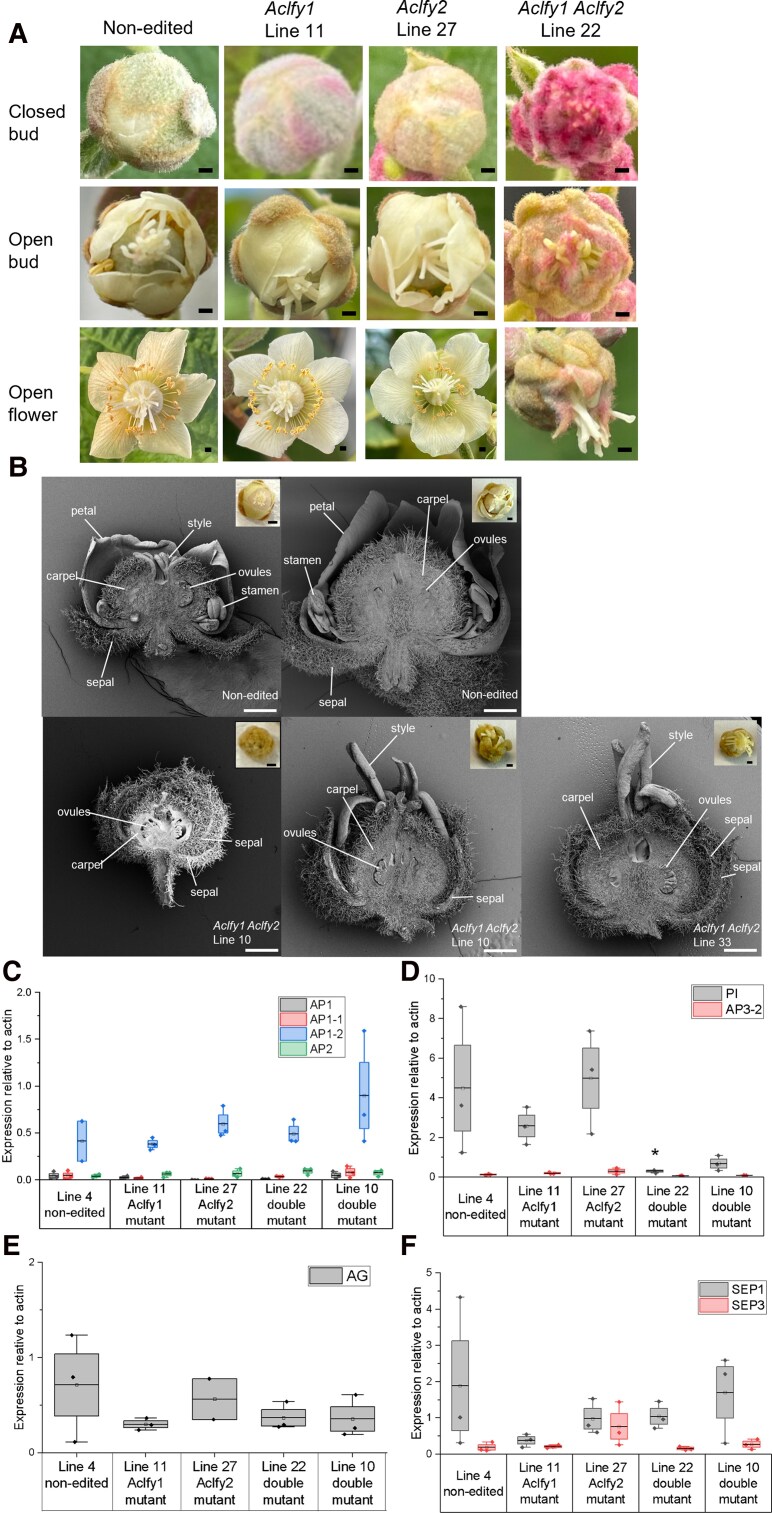
Abnormal floral development in kiwifruit *lfy1* and *lfy2* single-mutants, and in the *lfy1 lfy2* double-mutant. (A) Representative images of closed and open floral buds, and open flowers from the non-edited control (fast-flowering hermaphrodite background, *cen4 sygl* double-mutant), the *lfy1* and *lfy2* single-mutant lines 11 and 27, respectively, and the *lfy1 lfy2* double-mutant line 22 (Fig. 4). Scale bars are 1 mm. (B) SEM images of dissected floral buds from the non-edited control and the *lfy1 lfy2* double-mutant lines 10 and 33. Scale bars are 1 mm. The insets are images of the complete floral buds. (C–F) Relative expression of (C) the class A genes *AcAP1*, *AcAP1-1*, *AcAP1-2*, and *AcAP2*, (D) the class B genes *AcPI* and *AcAP3-2*, (E) the class C gene *AcAG*, and (F) the class E genes *AcSEP1* and *AcSEP3* in the floral buds of the non-edited, single-mutant, and double mutant plants. Expression is shown relative to that of the *ACTIN* reference gene. The plots show the mean (line inside box), the standard error of the mean (box), and the maximum and minimum data points data points (whiskers). Significant differences compared with the non-edited control were determined using two-sample *t*-tests: *P<0.05. Results using the alternative reference gene *EF1a* are shown in Supplementary Fig. S3.

In contrast, a striking difference in floral morphology was observed in the *lfy1 lfy2* double-mutants compared with the single-mutants and the non-edited controls (Fig. 5A, B). In the non-edited hermaphrodite floral buds, the expected four whorls of floral organs were present with prominent hairy sepals in the outer whorl (whorl 1), petals in whorl 2, stamens in whorl 3, and carpels in whorl 4 (Fig. 5A, B; Supplementary Fig. S3F). In contrast, SEM of the floral buds from the double-mutant lines 10 and 33 showed two whorls of sepals (whorls 1 and 2) but no petals (Fig. 5B). There were also no stamens, but the carpel in whorl 4 appeared to be intact with ovules similar to the non-edited lines. The double-mutant flowers also did not open as the non-edited flowers did (Fig. 5A, B). The double-heterozygote line, monoallelic gene-edited *lfy1*, and monoallelic gene-edited *lfy2* (line 18) produced flowers like the non-edited controls (Supplementary Fig. S3F). In summary, *lfy* single-mutants showed phenotypes comparable to controls whereas the *lfy1 lfy2* double-mutants exhibited a defective floral phenotype. The appearance of extra sepals, and absence of petals and stamens suggested that floral organ patterning might be affected by the knock out of the *LFY* genes in kiwifruit.

Therefore, we used RT-qPCR to investigate the expression of eight selected candidate kiwifruit ABCE class floral organ identity genes (Varkonyi-Gasic *et al.,* 2011, 2012; Ye *et al.,* 2022) in the closed floral buds from the various *lfy* mutants and non-edited lines (Fig. 5C–F; Supplementary Fig. S3A–E). There were no strong differences in the expression levels of the class A, C, and E genes tested. For the class B genes, *AP3-2* showed low overall expression, while the expression of *PI* was strongly reduced in the *lfy1 lfy2* double-mutant lines 22 and 10 (Fig. 5D; Supplementary Fig. S3B). This suggested that expression of *PI* was negatively affected by the lack of *LFY1* and *LFY2*, potentially contributing to the loss of petals and stamens in the double-mutant lines.

### Pollination of *lfy1 lfy2* double-mutant flowers results in non-viable seeds

**Fig. 6.**
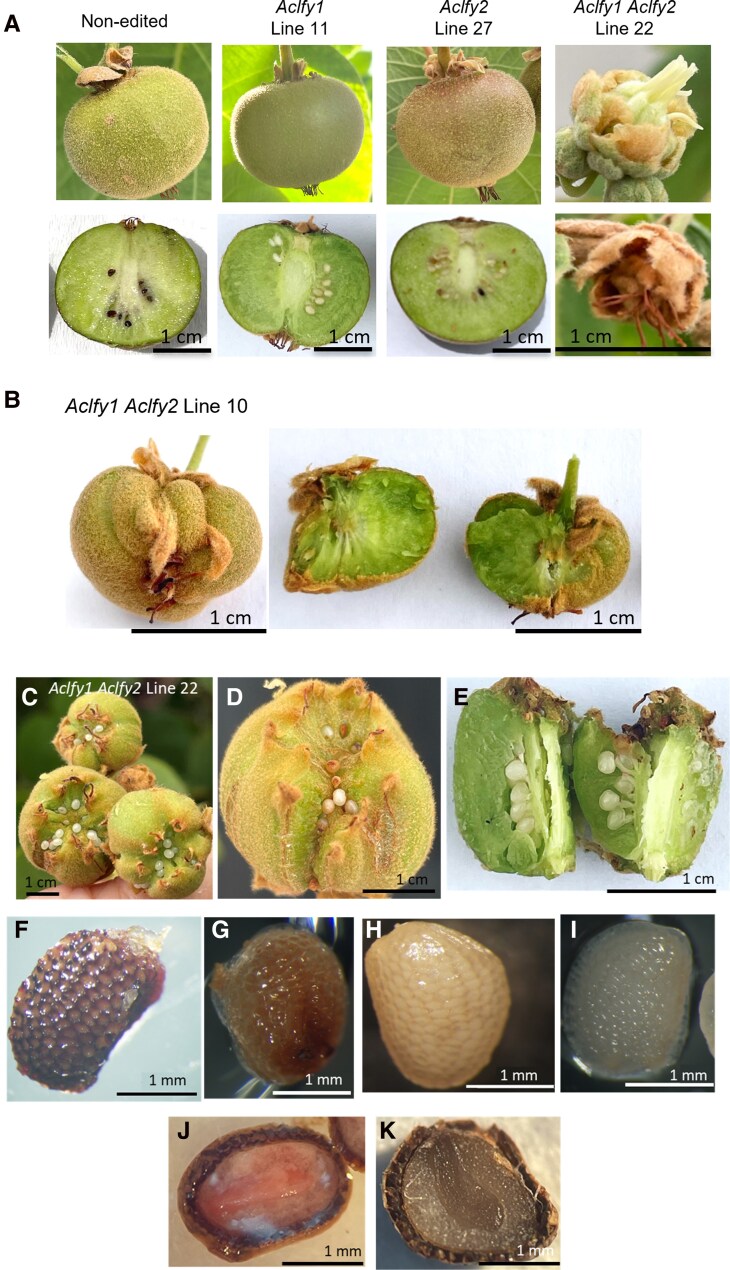
Cross-pollinated fruits from the kiwifruit *lfy1 lfy2* double-mutants in the fast-flowering hermaphrodite background have an abnormal shape and non-viable seeds. (A) Representative images of normal fruit developed from self-pollination of the non-edited line (*cen4 sygl* double-mutant) at 100 days after full bloom (DAFB), the *lfy1* single-mutant line 11 at 60 DAFB, and the *lfy2* single-mutant line 27 at 80 DAFB, together with desiccated flowers of the *lfy1 lfy2* double-mutant line 22, which never formed fruit. For details of the mutants see Fig. 4. (B) Fruit from cross-pollination using wild-type pollen in the *lfy1 lfy2* double-mutant line 10, displaying reduced size and no seeds. (C–E) Fruits from cross-pollination in the *lfy1 lfy2* double-mutant line 22 showing formed seeds exposed on the stylar end of the fruit as well as inside the fruit. (F) A mature dark seed inside the fruit of non-edited self-pollinated fast-flowering hermaphrodite kiwifruit and (G) a mature dark seed exposed on the stylar end of the fruit shown in (C). (H) An immature white seed inside the fruit of non-edited self-pollinated fast-flowering hermaphrodite kiwifruit and (I) an immature white seed exposed on the stylar end of the fruit shown in (C). (J, K) Tetrazolium staining of dark mature seeds showing (J) a viable but weak-vigour seed from a non-edited self-pollinated fruit and (K) a non-viable seed on the external stylar end of fruit from the *lfy1 lfy2* double-mutant line 22.

The fast-flowering hermaphrodite kiwifruit in which the *lfy1 lfy2* double-mutants were created are capable of self-pollination. The effect of their defective floral structure and arrangement on fruit development was investigated by examining the fruit formed from self- and cross-pollination events. The self-pollinated *lfy* single-mutants (lines 11 and 27) had wild-type-like fruit identical to those from the non-edited control plants (Fig. 6A). In contrast, the *lfy1 lfy2* double-mutant line 22 was unable to self-pollinate due to the lack of stamens, with the flowers eventually dying. Cross-pollination of the enclosed flowers of the double-mutant line 10 using wild-type pollen resulted in misshapen fruit (Fig. 6B), whilst cross-pollination of line 22 using wild-type pollen also resulted in misshapen fruit, with seeds exposed on the stylar end of the fruit (Fig. 6C, D). The style end of the fruit did not appear to fuse properly, and the seeds were embedded along the ridges of the unfused end. Seeds were also formed inside the fruit (Fig. 6E). Mature seeds from the self-pollinated non-edited controls were dark and characterised by hexagonal patterning on the surface visible under the microscope (Fig. 6F). The mature seeds from the exposed stylar end of the *lfy1 lfy2* double-mutant line 22 also eventually darkened and were characterised by the same surface pattern (Fig. 6G). Immature white seeds from inside the fruit and those exposed on the stylar end of the double-mutant fruit were of the same shape and again characterised by the same surface pattern (Fig. 6H, I). The mature dark seeds grown within the cross-pollinated fruit had a very low germination rate (1 in 29, Supplementary Fig. S4) and the seedling did not survive. Tetrazolium staining for viability suggested that the embryos of seed from the *lfy1 lfy2* double-mutant were non-viable, in contrast to the stained viable seed from self-pollinated control fruit (Fig. 6J, K).

### *LFY1* and *LFY2* promoters are differentially transactivated by SOC1 and WUS, and activation is strongly repressed by CEN4 in combination with FD2

**Fig. 7.**
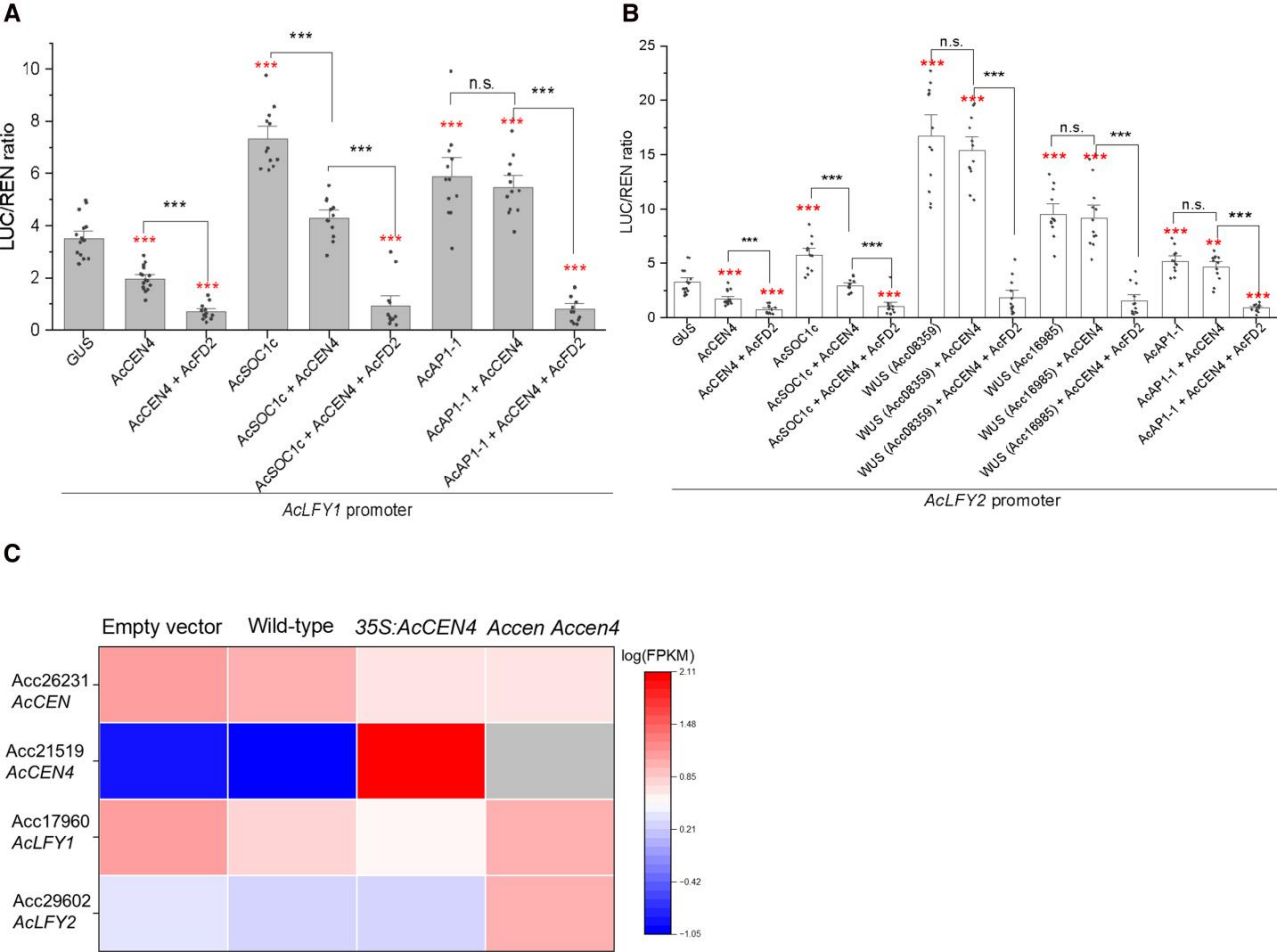
Kiwifruit *LFY1* and *LFY2* are regulated by CENTRORADIALIS (CEN) proteins. (A, B) Results of dual-luciferase assays in *Nicotiana benthamiana* leaves showing (A) *LFY1* promoter activation by SOC1c and AP1-1 and repression by co-infiltration of CEN4 and FD2 and (B) *LFY2* promoter activation by SOC1c, AP1-1, and WUS (Acc08359) and repression by co-infiltration of CEN4 and FD2. Data are means (±SEM) of four biological replicates from each experiment repeated at least three times. Significant differences between means were determined using two-sample *t*-tests; red asterisks indicate significant differences compared with the GUS control: **P<0.01; ***P<0.001; n.s., not significant. (C) Heat map of gene expression of *CEN*, *CEN4*, *LFY1*, and *LFY2* as determined by RNA-seq in shoot apices of the empty-vector control, the wild-type (‘Red9’), the *35S:AcCEN4-6×HA* line, and the *cen cen4* edited line. Details of expression levels from individual biological replicates are presented in Supplementary Table S6.

We next examined candidate factors that might regulate *LFY* gene expression in kiwifruit. In Arabidopsis, a SOC1–AGL24 protein complex binds to the *LFY* promoter to enhance expression (Lee *et al*., 2008) whilst in contrast *LFY* is repressed by the TFL1–FD complex (Zhu *et al*., 2020). To investigate whether aspects of *LFY* regulation are conserved in kiwifuit, we performed *cis*-motif analysis on the 2 kb promoter region upstream from the translation start site of *LFY1* and *LFY2* using the Plant ChIP-seq Database (PCBase 2.0; Chow *et al*., 2024). Transcription factor binding motifs including MYB, bHLH, bZIP, and SEP were identified in the promoters of both genes at different frequencies and positions (Supplementary Table S4; Supplementary Fig. S5A, B). Although the two promoters share 81% identity, the *cis*-motif for WUS was only found in the *LFY2* promoter. Interestingly, the exonic bZIP-binding motifs previously identified to be responsible for TFL recruitment (Zhu *et al*., 2020) were also present in both the *LFY* promoters 300–500 bp upstream of the translation start site. Dual-luciferase promoter transactivation assays in leaves of *N. benthamiana* demonstrated specific up-regulation with several AcSOC1-like and AcAP1-1 transcription factors (Fig. 7A, B) but not by the transcription factors AcFUL and AcSEP (Supplementary Fig. S5C–F). The WUS *cis*-motif and strong activation on only the *AcLFY2* promoter suggests a complex regulation of kiwifruit *LFY* expression (Supplementary Fig. S5B, F). In contrast, the combination of AcCEN4 and AcFD2 dramatically decreased the promoter activation of both *AcLFY1* and *AcLFY2* (Fig. 7A, B).

### CENs influence *LFY1* and *LFY2* expression in kiwifruit

To investigate the potential transcriptional regulation of *LFY* genes by CENs in kiwifruit flower development, we performed transcriptomic analysis by RNA-seq on shoot apices from *35S:CEN4-6×HA* and *cen cen4* edited lines and the corresponding empty-vector and the wild-type lines, respectively, all maintained in tissue-culture conditions (Supplementary Fig. S6A–G, Supplementary Table S5). The wild-type was transformed with *35S:CEN4-6×HA* to create *CEN4*-overexpression lines and *cen cen4* lines were generated using CRISPR-Cas9 mediated mutagenesis (Supplementary Tables S2, S6). The expression levels of *LFY1*, *LFY2*, *CEN*, and *CEN4* were examined in shoot apices and, as expected, the expression of *CEN4* was high in the overexpression lines, low in the wild-type and empty-vector control lines, and undetected in the *cen cen4* edited lines (Fig. 7C; Supplementary Table S6). *CEN* expression was comparatively higher in the empty-vector and wild-type shoot apices, and lower in the misexpression apices. *LFY1* was down-regulated in the *CEN4*-overexpression lines compared with the other lines (Fig. 7C, Supplementary Fig. S6F); however, it was expressed in the *cen cen4* lines at the same level as the controls (Supplementary Table S6). On the other hand, *LFY2* expression was not affected in the *CEN4*-overexpression lines, but it was up-regulated in the *cen cen4* lines. This suggested that the expression of both *LFY* genes in kiwifruit is influenced by the CENs, but not in an identical manner. Interestingly, a number of candidate floral organ identity genes such as *PI*, *AP3-2*, *AG*, and *UFO-like* were up-regulated along with *LFY2* in the shoot apices of the fast-flowering *cen cen4* lines (Supplementary Fig. S6F).

## Discussion

In this study we have identified and characterised two highly similar *LFY* paralogs, *LFY1* and *LFY2*, in the perennial vine kiwifruit (Fig. 1). While a single *LFY* gene has been reported in most plant species, the two in kiwifruit are probably due to whole-genome duplication events that occurred during *Actinidia* evolution (Shi *et al.,* 2010; Huang *et al*., 2013). Their high conservation at the protein level and their gene expression patterns suggest similar functional roles for the two genes. We found that both *LFY1* and *LFY2* are expressed during active growth in kiwifruit buds, suggesting a role in meristems. Their expression in axillary buds during growth in the spring and summer, no expression during the winter dormancy period, and subsequent expression in the second growing season was consistent with previous findings of the bimodal patterns of *LFY* expression over the two seasons that kiwifruit flowers develop (Walton *et al.*, 2001). This previously proposed role in the regulation of kiwifruit flowering was supported by expression of both *LFY* genes in flower buds; However, their additional expression in terminal buds that are non-floral indicated that they are more likely associated with regulation of vegetative and flower development rather than with floral induction.

Indeed, our examination of overexpression and knock out lines indicated that the *LFY* genes are not key determinants of flowering time in kiwifruit. While overexpression of the *AcLFY* genes in Arabidopsis promoted early flowering (Fig. 2), *LFY*-overexpression in kiwifruit did not affect flowering time and the genes were not required for precocious flowering of a fast-flowering hermaphrodite mutant (*cen4 sygl*; Fig. 3). While the fast-flowering background that we used might have obscured the effects of *lfy* mutations on floral induction, *LFY* is not always effective at accelerating flowering in other species either. For example, in apple (*Malus × domestica*), overexpression of the Arabidopsis *LFY* gene does not promote early flowering, but the plants have a columnar architecture with shortened internodes and short shoots, suggesting an alternative role in apple (Flachowsky *et al*., 2010). Overexpression of poplar *LFY* is inconsistent at promoting precocious poplar flowering and produces bushy phenotypes (Rottmann *et al*., 2000).

There were also changes to the vegetative phenotypes in the kiwifruit *LFY*-overexpression lines including serrated leaves (Fig. 3), similar to the lobed leaf margins observed in Arabidopsis when the *AtLFY* co-regulator *UFO* is overexpressed (Lee *et al.*, 1997). Overexpression of *LFY*s also led to increased axillary bud outgrowth in kiwifruit, indicating that the genes can contribute to shoot outgrowth in both the wild-type and fast-flowering hermaphrodite backgrounds, but are unable to alter the meristem fate (Fig. 3, Supplementary Fig. S1). *LFY* also promotes axillary meristem growth in Arabidopsis, branching in rice, and compound leaf development in legumes such as *Pisum sativum* (pea), *Medicago truncatula*, *Lotus japonicus*, and *Glycine max* (soybean) (Hofer *et al*., 1997; Bomblies *et al*., 2003; Rao *et al*., 2008; Wang *et al*., 2008, 2013; D. Wang *et al*., 2025; Moyroud *et al*., 2010; Chahtane *et al*., 2013). These functions have led to LFY being proposed to have an ancestral role of regulating cell division and meristem outgrowth in early land plants, predating the floral function in flowering plants (Tanahashi *et al.,* 2005; Moyroud *et al.*, 2010; Chahtane *et al.*, 2013). The fast-flowering kiwifruit mutants that we gene-edited do not branch, so potential loss of branching in *lfy1 lfy2* double-mutants would have to be determined in a wild-type genotype.

Combined mutation of *LFY1* and *LFY2* also resulted in aberrant floral phenotypes (Fig. 5; Supplementary Fig. S3), implicating *LFY*s in key redundant roles in floral patterning and floral organ development in kiwifruit. Arabidopsis class B gene mutants show conversion of petals to sepals and stamens to carpels (Bowman *et al*., 1989, 1991; Wuest *et al*., 2012). The kiwifruit *lfy1 lfy2* double-mutant floral phenotype was similar with no petals or stamens, and a second whorl of sepals (whorl 2) indicating a conversion of petals to sepals. It was not clear if there is an additional carpel whorl, but the misshapen unfused fruit arising from cross-pollination of the double-mutant might suggest this. Thus, the *LFY* genes appear to regulate kiwifruit floral patterning and floral organ development, particularly the organs in whorls 2 and 3, perhaps via affecting class B genes in a kiwifruit ABCE model, and possibly reflecting the proposed ancient link between *LFY* and class B genes established before angiosperm evolution (Moyroud *et al*., 2017). Consistent with this, the B gene *PI* showed reduced expression in the *lfy1 lfy2* double-mutant compared to the controls (Fig. 5D, Supplementary Fig. S3B). While floral patterning in wild-type kiwifruit male and female flowers is initially similar, because all four floral organs are initiated in both, the hermaphrodite background we used for gene-editing might have obscured later-acting sex-specific effects of the *LFY*s on floral development. In other plants such as legumes, *lfy* mutations also affect flower patterning and morphology, but not in identical ways. In pea, the *unifoliata* (*lfy*) mutant flower is also characterised by lack of petals and stamens, and consists of sepalloid and carpelloid floral organs and incomplete sepal whorls filled with bract-like laminae (Hofer *et al.*, 1997). On the other hand, in *M. truncatula*, *single leaflet1* mutants produce inflorescence-like structures instead of flowers (Wang *et al.*, 2008). The soybean *lfy1 lfy2* double-mutant produces clusters of leaf-like structures in place of floral organs (L. Wang *et al*., 2025). Gene-editing of poplar *LFY* also results in aberrant flowers, with underdeveloped leaf-like floral organs (Klocko *et al*., 2023).

The role of *LFY* in processes other than flowering time has been well examined, but many gaps in our knowledge remain about the regulation of its expression in non-model plants. We used promoter-sequence analysis and transactivation assays to study its regulation in kiwifruit, and our results suggested conservation in some of the regulatory pathways and divergence between the two kiwifruit *LFY* paralogs. For example, dual luciferase transactivation assays indicated that both the *LFY1* and *LFY2* promoters were activated by AP1-1 and SOC1c (Fig. 7). However, despite high homology between the two promoters, specific *cis*-motifs were found in each and differential activation with transcription factors was observed. For example, WUS transactivated only the *LFY2* promoter (Supplementary Fig. S5), but it is unclear at this stage if these interactions are necessary for proper flower-patterning in kiwifruit. Furthermore, our results suggest a conserved role of CEN in regulation of *LFY* genes. The floral repressor CEN4 in conjunction with the candidate partner FD2 strongly repressed the transactivation of the *LFY1* and *LFY2* promoters in our dual luciferase assays. RNA-seq analysis also suggested that the expression of the two *LFY* genes was negatively regulated by *CEN*s in kiwifruit but not in identical ways (Supplementary Fig. S6; Supplementary Table S6), suggesting a more complex regulation that was also indicated by the transactivation assays. In contrast to Arabidopsis *LFY* that is under dual and opposite regulation by TFL1 and FT (Collani *et al*., 2019; Zhu *et al*., 2020), the kiwifruit *LFY*s did not appear to be transcriptionally regulated by any combinations of FT and FD in the dual luciferase assays (Supplementary Fig. S5G, H).

In summary, our results suggest that *LFY1* and *LFY2* have overlapping functions in kiwifruit as key regulators of growth, development, and floral patterning, but not flowering time. This points to another similarity between the genes and pathways controlling flowering in annuals with those controlling growth and dormancy in kiwifruit (Voogd *et al.*, 2015, 2022; Ding and Nilsson, 2016; Wu *et al.*, 2017; Varkonyi-Gasic *et al.*, 2025); however, the phylogenetic position might also contribute to these differences. Overall, this study contributes to our fundamental understanding of the diversity of *LFY* function and highlights the complexity of flowering-time regulation in perennial species.

## Supplementary Material

erag092_Supplementary_Data

## Data Availability

The raw RNA-seq data are available in the NCBI Gene Expression Omnibus database (https://www.ncbi.nlm.nih.gov/geo/) under accession number GSE307710.

## Supplementary data

The following supplementary data are available at *JXB* online.

Fig. S1. Effects of overexpression of kiwifruit *LEAFY* genes in the wild-type background.

Fig. S2. Alignment of amino acid sequences from edited alleles of *LFY1* and *LFY2* in various single- and double-mutant lines.

Fig. S3. Gene expression in *lfy* mutants using *EF1a* as reference gene, and abnormal floral development.

Fig. S4. Seed germination test for the *lfy1 lfy2* double-mutant.

Fig. S5. *AcLFY1* and *AcLFY2* promoter *cis*-motifs and dual-luciferase promoter transactivation assays.

Fig. S6. Transcriptomic analysis of shoot apices of *CEN4*-overexpression and *cen cen4* edited lines.

Table S1. List of oligonucleotides used in this study.

Table S2. Gene-editing events for the *cen cen4* double-mutant lines used for RNA-seq.

Table S3. Number of nodes to flowering in the *lfy* single-mutant and the *lfy1 lfy2* double-mutant lines.

Table S4. *Cis*-motifs identified in *AcLFY1* and *AcLFY2* promoters

Table S5. Differentially expressed genes (DEGs) in the shoot apices of (A) *CEN4*-overexpression lines and (B) *cen cen4* edited lines compared with their controls, and (C) common DEGs between (A) and (B).

Table S6. Expression of selected genes implicated in flowering time regulation in *CEN4*-overexpression lines and *cen cen4* edited lines.
