# Supplementary material for: Two LEAFY homologs regulate floral patterning and development without affecting flowering time in kiwifruit
Source: J Exp Bot. 2026 Feb 19;77(12):3740–57. doi: 10.1093/jxb/erag092 (PMC13293076; doi:10.1093/jxb/erag092)
Supplement: erag092_Supplementary_Data [file erag092_supplementary_data.zip › erag092 NEW - supplementary figures_06.03.26.pdf]

Supplementary Fig. S1

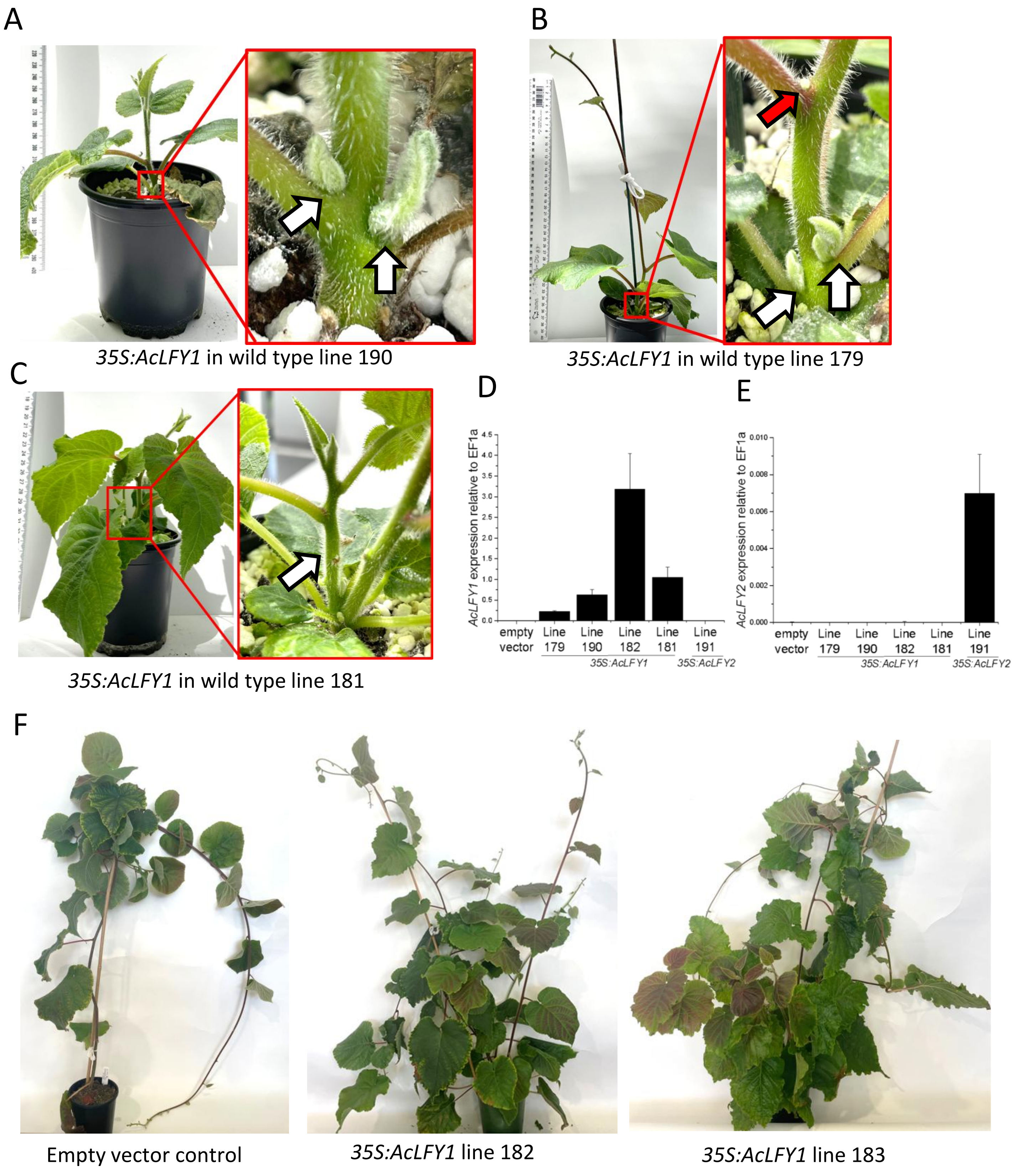

Supplementary Figure S1. Overexpression of *AcLFY1* induced axillary shoot initiation in wild-type background. (A – C) Three independent lines overexpressing *AcLFY1* showed lateral shoot outgrowth at the lower nodes. Red box highlights the internode space on the primary shoot for examination in close up, red arrow represents activated budbreak and white arrow represents lateral shoot outgrowth. (D) Expression of *AcLFY1* and *AcLFY2* in independent *AcLFY1* or *AcLFY2* overexpression lines. (F) Multiple secondary shoots from the lines overexpressing *AcLFY1* compared to the single stem empty vector control.

Supplementary Fig. S2

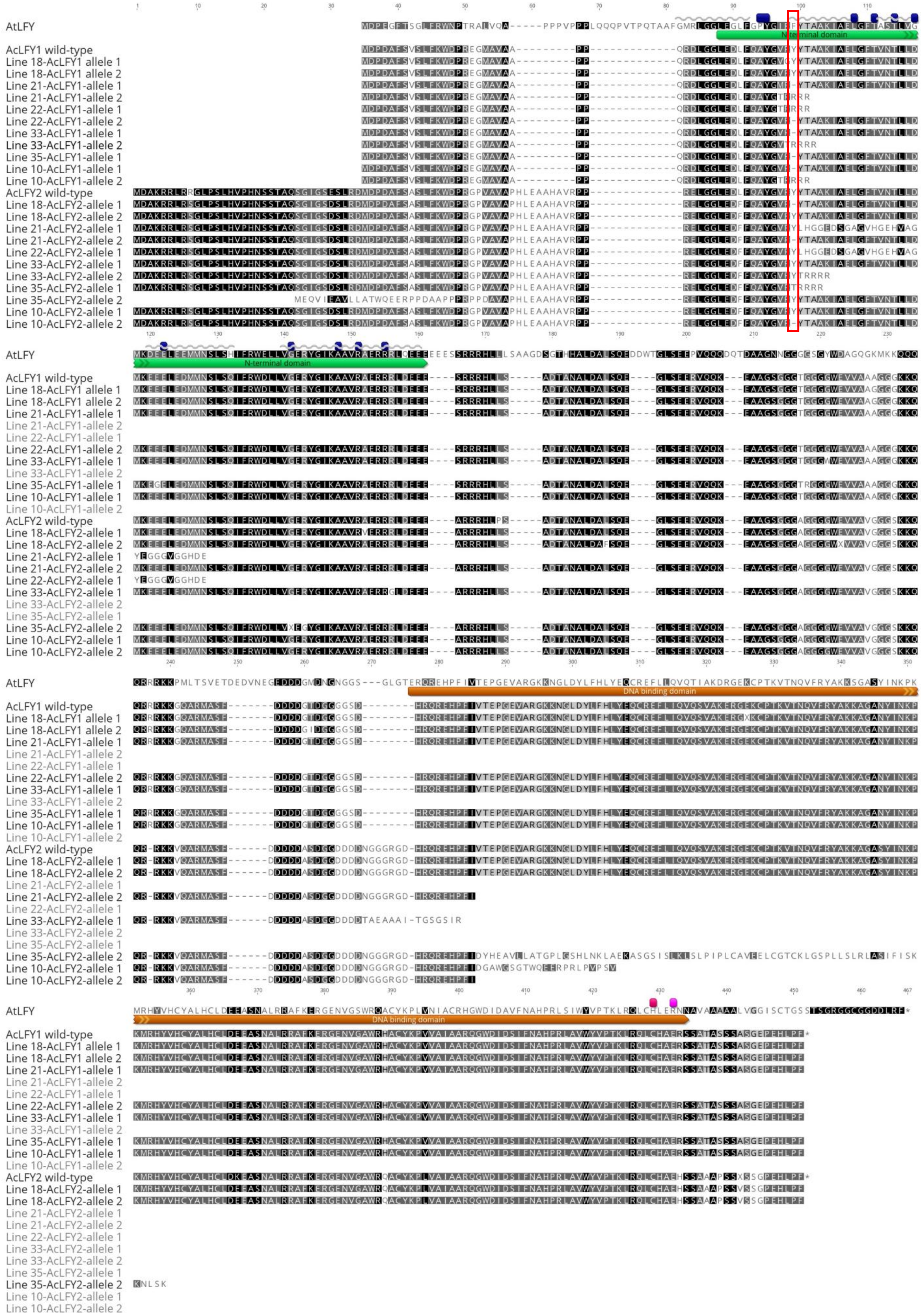

Supplementary Figure S2. Alignment of predicted amino acid sequences translated from edited alleles of *AcLFY1* and *AcLFY2* from some of the single and double mutant lines. Alpha helices indicated by the coil annotations. Blue boxes on top on the sequence indicate the key interaction residues of the sterile Alpha Motif oligomerisation domain in the N-terminal domain. Red box indicates the edit induced by guide 1 in exon 1 caused either a frameshift mutation or a three nucleotide deletion that resulted in a single amino acid deletion which is the first residue of the second alpha helix structure.

Supplementary Fig. S3

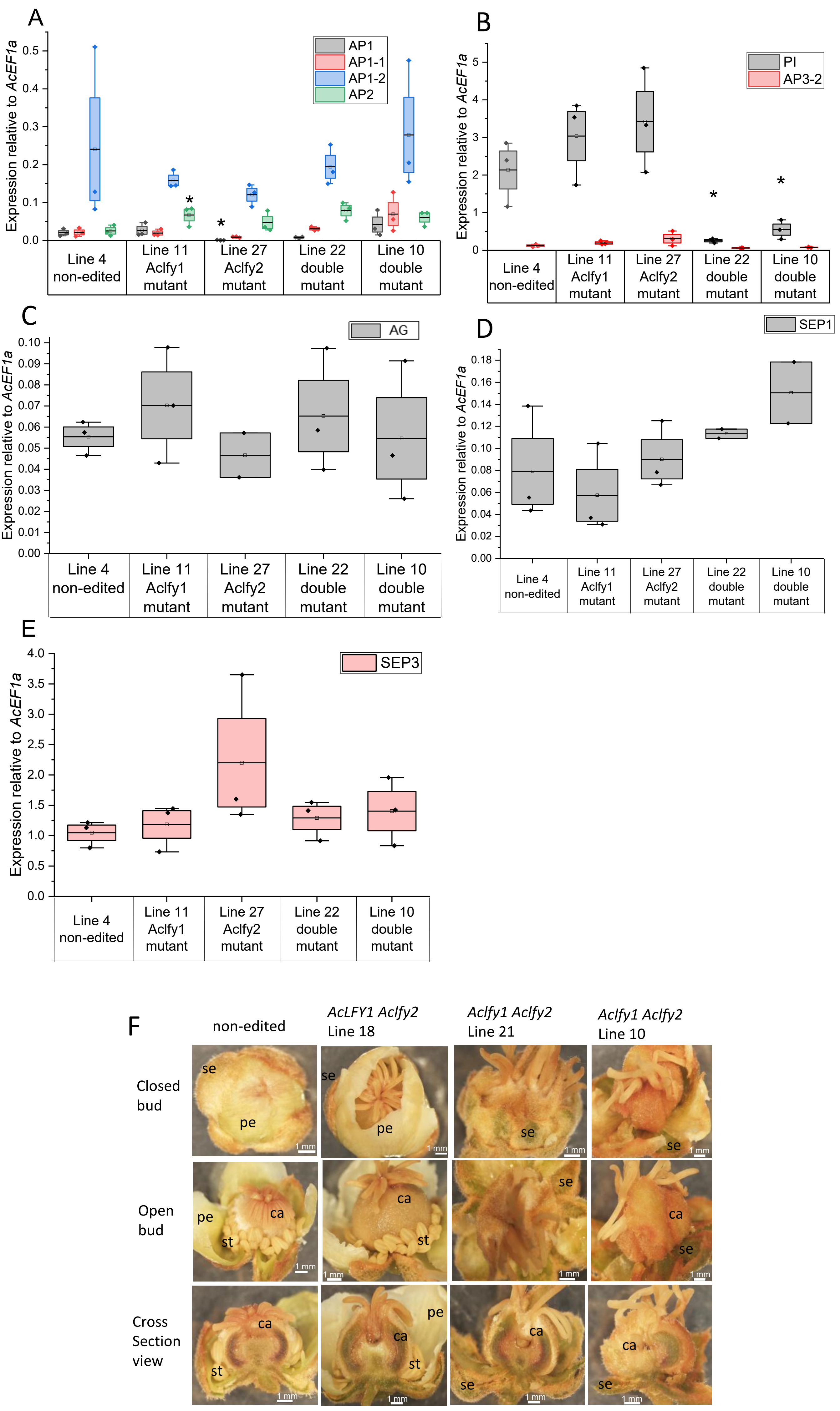

Supplementary Figure S3. Gene expression in *lfy* mutants using *EF1a* as reference gene, and abnormal floral development in double mutants. (A) Gene expression of class A genes *AcAP1*, *AcAP1-1*, *AcAP1-2*, and *AcAP2*, class B genes *AcPI* and *AcAP3-2* (B), class C gene, *AcAG* (C), and class E genes *AcSEP1* (D) and *AcSEP3* (E) in the non-edited, single mutant and double mutant floral buds. Mean is shown as the middle line and whiskers are maximum and minimum data points. Range of the box indicates the standard error of the mean. The Two-Sample t-Test was used to determine if the *Aclfy* mutant mean value was significantly different from the non-edited control: $p < 0.05$*. (F) The floral buds from the non-edited, single *Aclfy2* biallelic gene edited mutant line 18 and the double *Aclfy1 Aclfy2* biallelic gene edited mutant line 21 and line 10 dissected under the stereomicroscope. Se: sepal, pe: petal, st: stamen, ca: carpel. Scale bar = 1 mm.

Supplementary Fig. S4

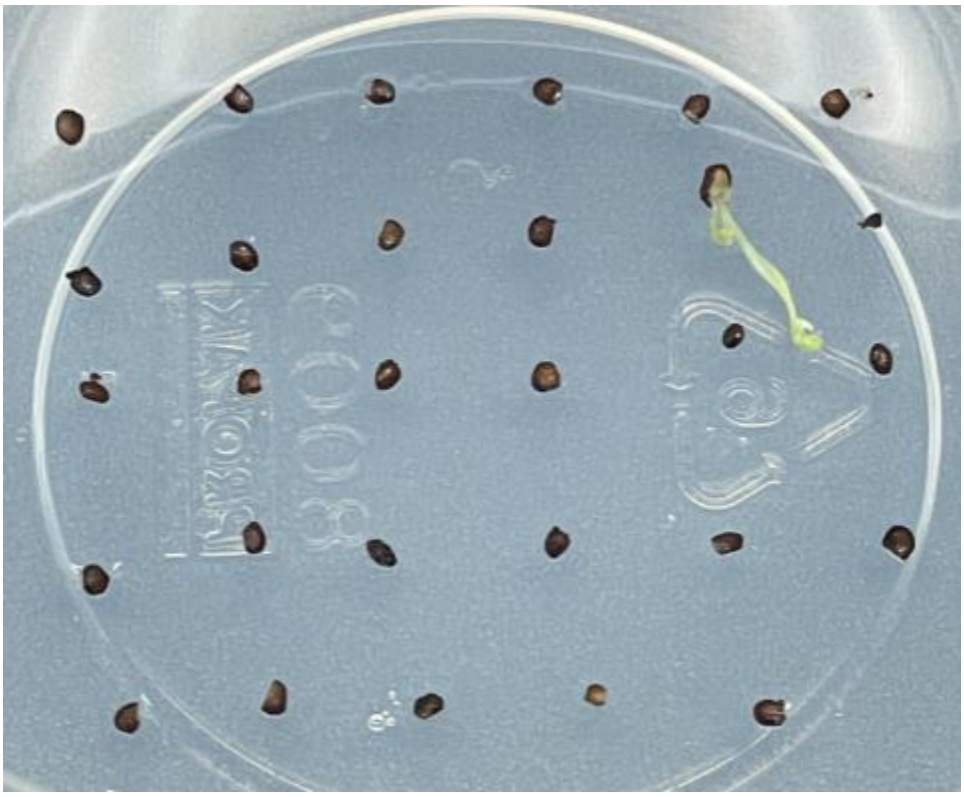

Supplementary Figure S4. Germination test of the mature dark seeds from inside the fruit of the *Aclfy1 Aclfy2* double mutant line 22.

Supplementary Fig. S5

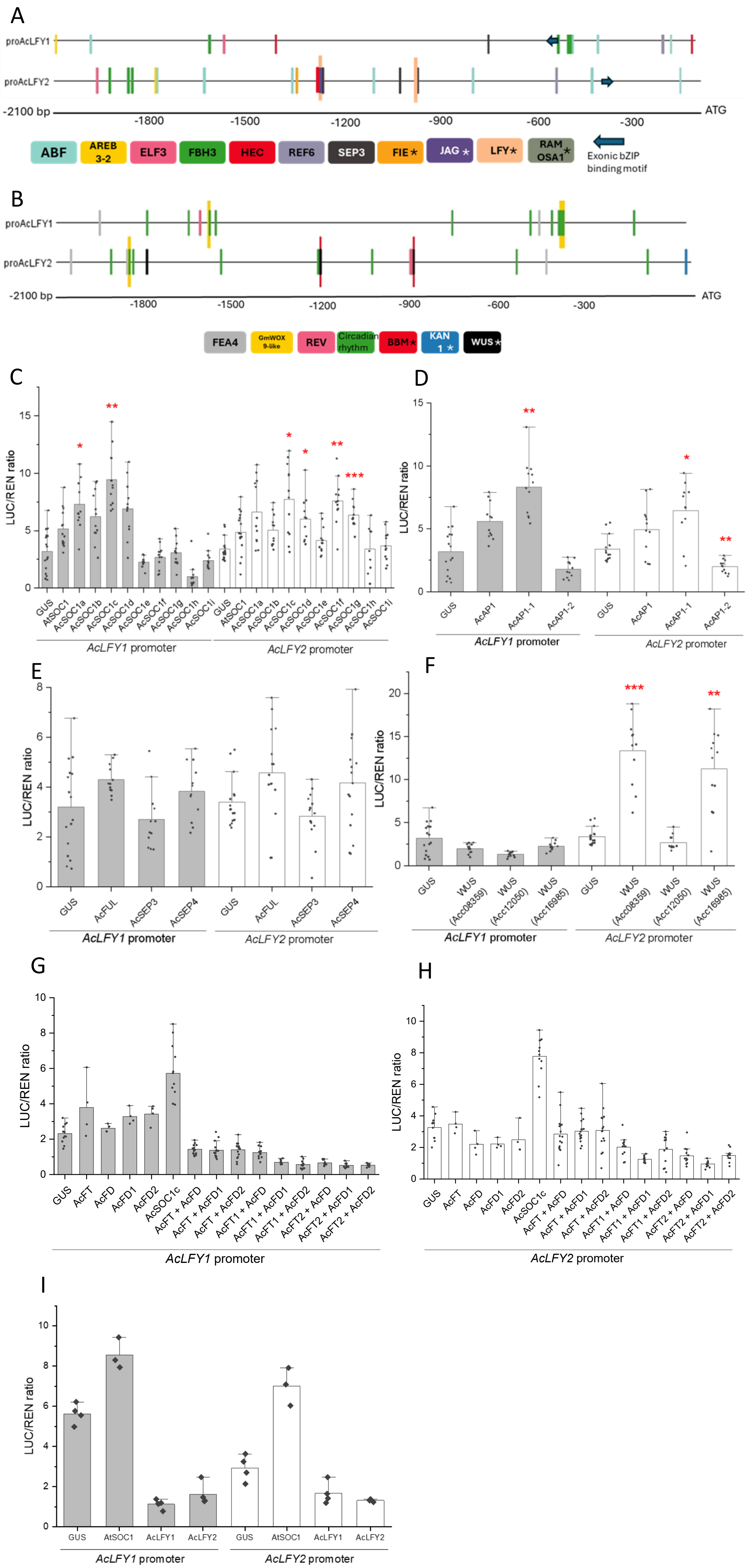

Supplementary Figure S5. *AcLFY1* and *AcLFY2* promoters harbour the regulatory cis-motifs and are activated by several AcSOC1. (A) Presence of flowering-related binding motifs identified in the *AcLFY1* and *AcLFY2* promoters as predicted by the ChIP-seq database (PCBase2.0). The motifs labelled with asterisks are only found in *AcLFY2* promoter. (B) Presence of meristem regulatory binding motifs identified in the *AcLFY1* and *AcLFY2* promoters as predicted by the ChIP-seq database (PCBase2.0). The motifs labelled with asterisks are only found in *AcLFY2* promoter. All the presented motifs have HIT score of 1. (C) Dual luciferase transactivation assay showing *AcLFY1* and *AcLFY2* promoters activation by the Arabidopsis AtSOC1, kiwifruit AcSOC1a – I compared to GUS control, (D) AcAP1, AcAP1-1, AcAP1-2 compared to GUS control, (E) AcFUL, AcSEP3, AcSEP4 compared to GUS control, (F) WUS gene models (Acc08359, Acc12050 and Acc16985) compared to GUS control. (G) *AcLFY1* promoter activation by combinations of AcFT and AcFD. (H) *AcLFY2* promoter activation by combinations of AcFT and AcFD. (I) *AcLFY1* and *AcLFY2* promoter activation by AcLFY1 and AcLFY2. Data were shown as means ± SEM of four biological replicates from each experiment repeated three times. One-way ANOVA with a significance level (α) of 0.05 was performed to confirm statistical significance against the GUS control: $p < 0.05$*, $p < 0.01$**, and $p < 0.001$***.

Supplementary Fig. S6

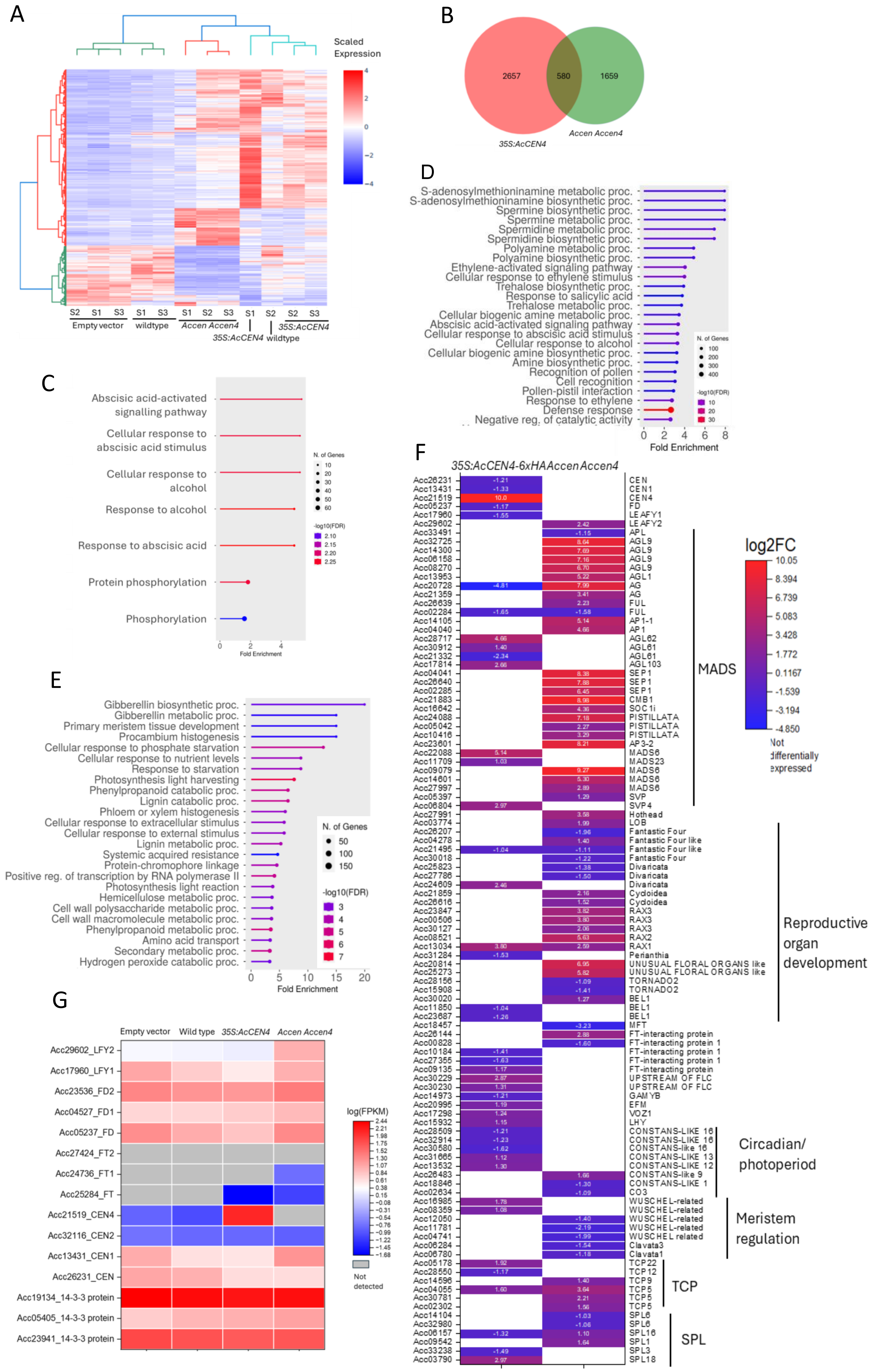

Supplementary Figure S6. Transcriptomic analysis of *35S:AcCEN4-6xHA* shoot apices and *Accen/cen4* edited shoot apices compared with the empty vector control and wild type, respectively. (A) Hierarchical clustering of gene expression (FPKM) of differentially expressed genes (DEGs) in empty vector, wild-type, *35S:AcCEN4-6xHA,* and *Accen/cen4* edited shoot apices. (B) Overlap and non-overlap of DEGs between the *35S:AcCEN4-6xHA* and *Accen/cen4* shoot apices. (C) GO enrichment analysis of the differentially expressed genes shared between the *35S:AcCEN4* and *Accen/cen4* edited shoot tips. Top 20 pathways shown out of a total of 80 pathways, selected by FDR cutoff 0.01 and sorted by fold enrichment.(D) Gene ontology (GO) enrichment analysis of the differentially expressed genes (DEGs) exclusive in the *35S:AcCEN4-6xHA* shoot apices. Top 20 pathways shown out of a total of 80 pathways, selected by FDR cutoff 0.01 and sorted by fold enrichment. (E) GO enrichment analysis of the DEGs exclusive in the *Accen/cen4* edited shoot apices. Top 20 pathways shown out of a total of 80 pathways, selected by FDR cutoff 0.01 and sorted by fold enrichment. (F) Log2FC expression of the flowering-related candidate DEGs from 35S:AcCEN4-6xHA shoot apices compared to the empty vector shoot apices and Accen/cen4 edited shoot apices compared to the wild-type shoot apices. (G) Gene expression (FPKM) heatmap of the key genes in the Flowering-Activation-Complex (FAC) and *AcLFY1* and *AcLFY2*.
